# Arabidopsis AtMORC4 and AtMORC7 Form Nuclear Bodies and Repress a Large Number of Protein-Coding Genes

**DOI:** 10.1371/journal.pgen.1005998

**Published:** 2016-05-12

**Authors:** C. Jake Harris, Dylan Husmann, Wanlu Liu, Farid El Kasmi, Haifeng Wang, Ashot Papikian, William A. Pastor, Guillaume Moissiard, Ajay A. Vashisht, Jeffery L. Dangl, James A. Wohlschlegel, Steven E. Jacobsen

**Affiliations:** 1 Department of Molecular, Cell and Developmental Biology, University of California at Los Angeles, Los Angeles, California, United States of America; 2 Department of Biology, University of North Carolina at Chapel Hill, Chapel Hill, North Carolina, United States of America; 3 Basic Forestry and Proteomics Research Center, Haixia Institute of Science and Technology (HIST), Fujian Agriculture and Forestry University, Fuzhou, Fujian, China; 4 Fujian Province Key Laboratory of Plant Virology, Institute of Plant Virology, Fujian Agriculture and Forestry University, Fuzhou, Fujian, China; 5 Department of Biological Chemistry, David Geffen School of Medicine, University of California, Los Angeles, Los Angeles, California, United States of America; 6 Howard Hughes Medical Institute, University of North Carolina at Chapel Hill, Chapel Hill, North Carolina, United States of America; 7 Howard Hughes Medical Institute, University of California at Los Angeles, Los Angeles, California, United States of America; Edinburgh Cancer Centre, UNITED KINGDOM

## Abstract

The MORC family of GHKL ATPases are an enigmatic class of proteins with diverse chromatin related functions. In Arabidopsis, AtMORC1, AtMORC2, and AtMORC6 act together in heterodimeric complexes to mediate transcriptional silencing of methylated DNA elements. Here, we studied Arabidopsis *AtMORC4* and *AtMORC7*. We found that, in contrast to AtMORC1,2,6, they act to suppress a wide set of non-methylated protein-coding genes that are enriched for those involved in pathogen response. Furthermore, *atmorc4 atmorc7* double mutants show a pathogen response phenotype. We found that AtMORC4 and AtMORC7 form homomeric complexes *in vivo* and are concentrated in discrete nuclear bodies adjacent to chromocenters. Analysis of an *atmorc1*,*2*,*4*,*5*,*6*,*7* hextuple mutant demonstrates that transcriptional de-repression is largely uncoupled from changes in DNA methylation in plants devoid of MORC function. However, we also uncover a requirement for MORC in both DNA methylation and silencing at a small but distinct subset of RNA-directed DNA methylation target loci. These regions are characterized by poised transcriptional potential and a low density of sites for symmetric cytosine methylation. These results provide insight into the biological function of MORC proteins in higher eukaryotes.

## Author Summary

Keeping selfish genetic elements–such as transposons–silent, while maintaining access to genes, is a fundamental challenge for eukaryotes. Different pathways frequently converge in order to identify transposons and maintain their repression, and in *Arabidopsis thaliana*, transposons are marked with DNA methylation. Previous studies of the Arabidopsis MORC proteins, which represent a highly conserved protein family, showed that AtMORC1, AtMORC2, and AtMORC6 are required for repression of methylated target transposons. Here, we describe the Arabidopsis genes *AtMORC4* and *AtMORC7*, which, instead of targeting methylated elements, appear to act redundantly to repress a large set of protein-coding genes and are required to mount a full defense against pathogen challenge. These proteins localize throughout the nucleus and form punctate bodies at the boundaries of highly compacted chromatin. By knocking out all functional copies of MORC genes in *Arabidopsis*, we find that major changes in transcription are not generally associated with the loss of DNA methylation. However, MORC may be recruited to assist in silencing of methylated regions that are unusually susceptible to transcriptional re-activation. This indicates that MORC and DNA methylation are convergently required to maintain repression at transposon targets.

## Introduction

Maintaining regulatory access to genes while repressing the expression of potentially deleterious transposable elements is a fundamental challenge for living organisms. Eukaryotes achieve this in part by parsing their genomes into functional units characterized by distinct chromatin features [1,2]. The most stable chromatin mark is cytosine DNA methylation [3]. In plants, DNA methylation is often associated with transcriptionally silent regions [4,5] and occurs primarily in three sequence contexts, CG, CHG and CHH (where H is defined by any base except G). Methylation at the symmetrical CG and CHG sites is maintained by the action of MET1—the homologue of mammalian DNMT1—and CMT3, respectively [6]. Asymmetric CHH methylation must be continuously re-established. In pericentromeric heterochromatin, this is mostly mediated by CMT2 [7,8]; while in small patches of heterochromatin in the otherwise euchromatic arms, CHH methylation is mostly maintained by the action of DRM2 in the RNA-directed DNA methylation (RdDM) pathway [9–11].

RdDM primarily targets transposable elements through the combined action of two plant specific RNA polymerases [12,13]. During RdDM, Polymerase IV (Pol IV) is in part recruited by SHH1 [14] to generate short transcripts [15–17], which are made double-stranded by the action of RDR2 and diced into 24nt small RNAs by DCL3. Polymerase V (Pol V) is targeted to methylated sites via SUVH2/9 [18,19] and generates scaffold transcripts to recruit 24nt small RNA directed complexes [20,21], which then recruit the *de novo* methyltransferase DRM2 to induce DNA methylation in all sequence contexts [10]. The RdDM pathway results in a robust self-reinforcing loop; however, a potential role for 21nt small RNAs and RDR6 during the early stages of methylation establishment has recently emerged [22–24].

To identify novel factors involved in transcriptional gene silencing, forward genetic screens from three independent laboratories isolated alleles of *AtMORC6* [NP_173344; *AT1G19100; CRH6; Defective in Meristem Silencing 11 (DMS11)*] [25–27]. MORC proteins are members of the GHKL ATPase superfamily [28,29] and by evolutionary comparison with prokaryotes are predicted to play a role DNA superstructure manipulations in response to epigenetic signals [30]. While the involvement of AtMORC6 in transcriptional repression is established, the extent to which it contributes to DNA methylation at target loci has varied between reports [25–27]. For instance, a 2012 study [25] found little evidence for methylation changes at either the de-repressed reporter construct or genome wide, while Lorković et al., 2012 [26] and Brabbs et al., 2013 [27] both observed minor reductions in DNA methylation at their reporter loci. It therefore remains uncertain whether transcriptional activation is associated with loss of DNA methylation in *atmorc* mutants and to what extent AtMORC proteins are involved in the RdDM pathway.

Another member of the *A*. *thaliana* MORC family, *AtMORC1* [NP_568000; AT4G36290; *Compromised Recognition of Turnip Crinkle Virus 1 (CRT1)*], is involved in plant defense and was isolated as a mutant that is hyper-sensitive to Turnip Crinkle Virus [31]. Interestingly, *AtMORC1* was also identified in the same transcriptional repression screen that isolated *AtMORC6* [25]. Recent studies have implicated changes in DNA methylation and transcriptional responses to pathogen infection [32–34]. Yet it is unclear how AtMORC1 might function in both plant defense and transcriptional repression at RdDM targets. AtMORC1 and its very close homolog AtMORC2 act in mutually exclusive heteromeric complexes with AtMORC6, and an *atmorc1 atmorc2 atmorc6* triple mutant resembles that of *atmorc6* with regard to transcriptional profile and methylation state [35].

As there are seven members of the MORC family in Arabidopsis, we sought to characterize the remaining *AtMORC* genes in order to help elucidate MORC function. We found that the highly related AtMORC4 [NP_199891; AT5G50780; *CRH4*] and AtMORC7 [NP_194227; AT4G24970; CRH3] proteins act partially redundantly to transcriptionally repress a large regulon and also play a role in plant defense. Both AtMORC4 and AtMORC7 were found to form stable homomers, but do not interact with each other, suggesting that they act in parallel to control gene silencing. We also found that AtMORC4 and AtMORC7, like AtMORC1 and AtMORC6 [25], form nuclear bodies that are adjacent to chromocenters. Finally, by generating a compound mutant devoid of all MORC function, we demonstrate that transcriptional de-repression can be largely uncoupled from changes in DNA methylation. However, a small but distinct subset of RdDM loci that are poised for transcriptional reactivation exhibit MORC-dependent methylation changes and reduced symmetric methylation potential.

## Results and Discussion

### AtMORC4 and AtMORC7 act semi-redundantly at a common set of loci

**Fig 1:**
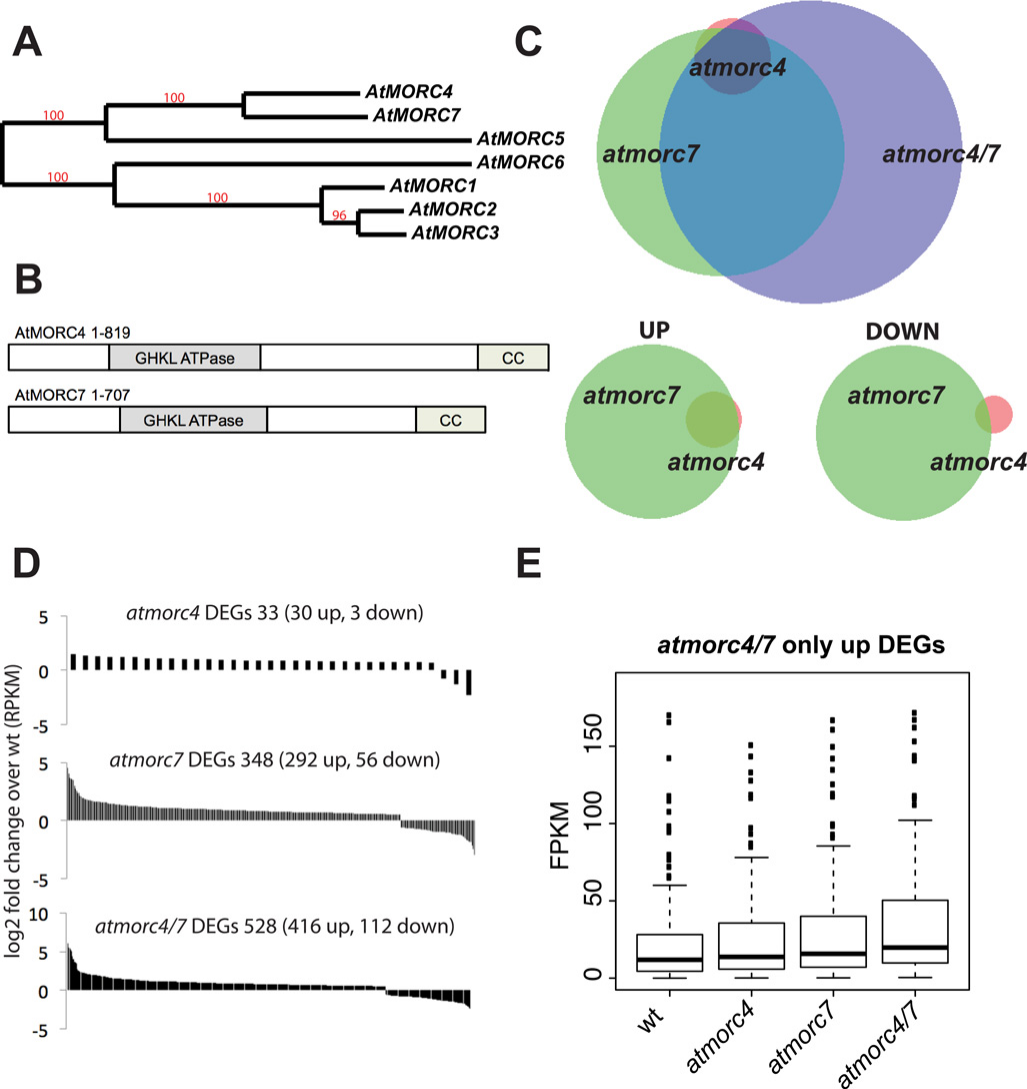
AtMORC4 and AtMORC7 act in a partially redundant manner to repress a highly overlapping gene set. **(A)** Phylogenetic reconstruction of *Arabidopsis thaliana AtMORC* genes (genomic sequence). Red numbers indicate branch support values in percentage (http://www.phylogeny.fr). **(B)** Schematic representation of AtMORC4 and AtMORC7 domains, drawn approximately to scale (CC = coiled coil). **(C)** Upper: Overlap of differentially expressed genes (DEGs–includes both genes and transposons—FDR<0.05) in the mutants indicated Lower: overlap of *atmorc4* and *atmorc7* in either upregulated (UP) or downregulated (DOWN) loci. There is greater overlap for the upregulated loci. Within each overlap, circle size and overlap is proportional to number of DEGs therein **(D)** log_2_ fold change for individual DEGs in each of the mutants indicated (ranked highest to lowest). Most are upregulated. **(E)** FPKM (fragments per kilobase per million reads) boxplots for upregulated DEGs only present in *atmorc4/7*, showing that the *atmorc4* and *atmorc7* single mutants also show a similar trend at these loci.

*AtMORC4* and *AtMORC7* are highly related to one another (Fig 1A and 1B) [35]. We obtained T-DNA knockout lines for these genes (*atmorc4-1* and *atmorc7-1*) (S1A Fig). RT-PCR at targets known to be de-repressed in the *atmorc6* background [25,35] showed little change in transcript levels in the homozygous knockouts. However, when we crossed the lines to create an *atmorc4-1 atmorc7-1* double knockout, we observed de-repression at several of the candidate loci, suggesting that AtMORC4 and AtMORC7 act redundantly (S1B Fig). To determine the extent of redundancy between AtMORC4 and AtMORC7, we performed mRNA-Sequencing (RNA-seq) on leaves from individual plants of Col-0, *atmorc4-1*, *atmorc7-1*, and *atmorc4-1 atmorc7-1* backgrounds (hereafter referred to as wild-type (wt), *atmorc4*, *atmorc7* and *atmorc4/7*, respectively). We found that AtMORC4 and AtMORC7 affect a highly overlapping gene set with AtMORC7 playing a more dominant role (Fig 1C–1E). In *atmorc7*, 348 annotated loci were differentially expressed (FDR < 0.05) with 84% being up-regulated. In *atmorc4*, the 33 differentially expressed loci (30 up, 3 down) were largely a subset of those altered in *atmorc7*, with 29 of the 30 up-regulated loci also up-regulated in *atmorc7*. In the *atmorc4/7* double knockout, 50% more loci were differentially expressed than in the individual knockouts combined, suggesting a significant level of redundancy between AtMORC4 and AtMORC7. Taken together, the results suggest that AtMORC4 and AtMORC7 act in a partially redundant manner, with AtMORC7 having a stronger effect than AtMORC4, to mainly repress a highly overlapping gene set.

### AtMORC4 and AtMORC7 form homomeric complexes *in vivo*

**Fig 2:**
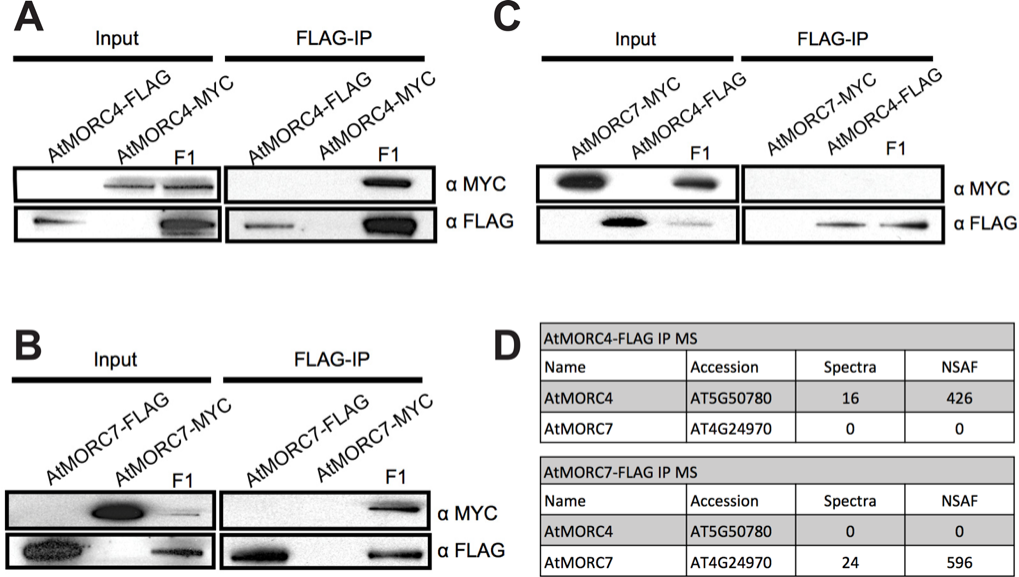
AtMORC4 and AtMORC7 form homomeric complexes *in vivo*. **(A)** Co-immunoprecipitation of AtMORC4-MYC with AtMORC4-FLAG in F1 plants **(B)** Co-immunoprecipitation of AtMORC7-MYC with AtMORC7-FLAG in F1 plants. **(C)** No interaction by co-immunoprecipitation between AtMORC7-MYC and AtMORC4-FLAG in F1 plants. **(D)** Table from immunoprecipitation followed by mass spectrometry (IP-MS) of FLAG tagged AtMORC4 and AtMORC7 plants showing peptides from themselves but not each other. NSAF = normalized spectral abundance factor.

We have previously shown that AtMORC6 forms mutually exclusive heteromeric complexes with either AtMORC1 or AtMORC2 [35]. To assess whether AtMORC4 and AtMORC7 form heteromeric complexes, we generated endogenous promoter driven MYC or FLAG tagged lines for both AtMORC4 and AtMORC7 in their respective T-DNA backgrounds. By co-immunoprecipitation, we detected a homotypic association of AtMORC4 and AtMORC7 but did not detect heteromers (Fig 2A–2C). These results were confirmed by mass spectrometry of the immunoprecipitated samples (IP-MS), showing that the AtMORC4 and AtMORC7 precipitates do not contain peptides from AtMORCs other than themselves (Fig 2D). Together, this indicates that AtMORC4 and AtMORC7 form homomeric complexes *in vivo*, consistent with the genetic redundancy observed between them (see Fig 1, S1 Fig).

### Transcriptome comparison between *AtMORC* knockouts

To directly compare the phenotypes of the *atmorc4* and *atmorc7* mutants with the previously characterized *atmorc6-3* (hereafter referred to as *atmorc6*), we performed a second round of RNA-seq analysis. We also sought to generate a genetically *MORC-*less plant to obtain an unobfuscated view of MORC function. For this, we created a higher order knockout plant containing T-DNA inserts in six out of the seven *MORC* genes in Arabidopsis, *atmorc1-2*, *atmorc2-1*, *atmorc4-1*, *atmorc5-1*, *atmorc6-3*, and *atmorc7-1* (*atmorc1/2/4/5/6/7*). While a previous study reported embryonic lethality for a T-DNA insertion in *AtMORC3* [NP_195350; AT4G36270; CRH2] [36], it is likely that this is an indirect effect caused by an unknown linked mutation in the SALK line (SALK_000009), as we found evidence suggesting that *AtMORC3* is in fact a pseudogene (S2 Fig). We found a premature stop codon in exon three in Col-0 (causing either an un-translated or truncated protein). Additionally, an independent homozygous T-DNA allele (SALK_043244) with an exonic insertion exhibited no discernable phenotype. Given that *AtMORC3* is non-functional in Col-0, the *atmorc1/2/4/5/6/7* line effectively lacks any functional AtMORC protein.

**Fig 3:**
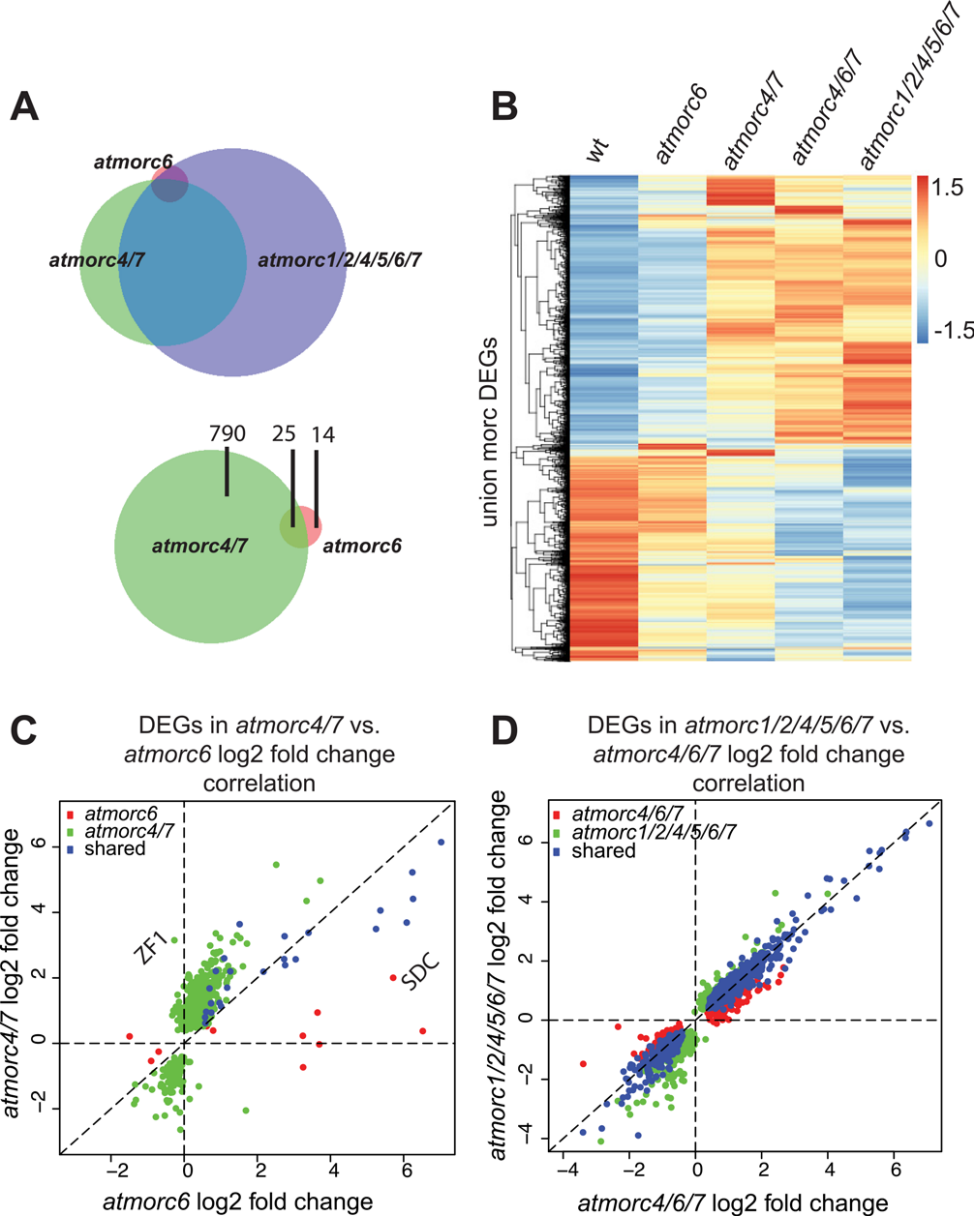
AtMORC4 and AtMORC7 target a wide gene set. **(A)** Upper: overlap of DEGs in the genotypes indicated with circle size and overlap proportional to number of DEGs therein. Lower: overlap between *atmorc4/7* and *atmorc6* DEGs, with number of DEGs indicated. **(B)** Heatmap over the union set of DEGs (FDR<0.05) in the different genotypes. Each row is normalized by z-score (red = relatively higher, blue = relatively lower expression in that genotype). **(C)** Correlation between *atmorc4/7* and *atmorc6* DEGs. *ZF1* and *SDC* are indicated as examples of loci specifically upregulated in *atmorc4/7* or *atmorc6*, respectively. **(D)** Correlation between *atmorc4/6/7* and *atmorc1/2/4/5/6/7* DEGs.

RNA-seq on individual plants (3 replicates each) from *atmorc6*, *atmorc4/7*, *atmorc4/6/7*, and *atmorc1/2/4/5/6/7* revealed 39, 815, 1188, and 1519 differentially expressed genes (FDR < 0.05) relative to wt, respectively, with a variety of interesting features (Fig 3). Twenty times more loci were differentially expressed in *atmorc4/7* as compared to *atmorc6*, suggesting that AtMORC4 and AtMORC7 play a more central role in gene expression (Fig 3A). As the majority of these *atmorc4/7* differentially expressed genes were up-regulated (87%), this is consistent with a repressive role and direct regulation at these targets. However, we cannot exclude the possibility of indirect effects. The difference between *atmorc6* and *atmorc4/7* is also clearly apparent from a heatmap over the union set of differentially expressed loci, which shows that *atmorc6* is most similar to wt (Fig 3B). In *atmorc6*, transposable elements (TEs) constitute 29% (11 total) of the differentially expressed loci while in *atmorc4/7*, only 1% (9 total) were misregulated, suggesting that AtMORC6 is preferentially involved in TE repression while AtMORC4 and AtMORC7 are primarily responsible for the repression of protein-coding genes.

Comparing *atmorc4/7* to *atmorc6* revealed that while there was a generally positive correlation, many loci are specifically affected in either *atmorc6* or *atmorc4/7* (Fig 3C). One example is *ZF1*, which encodes a stimulus response zinc finger protein characteristic of the types of genes up-regulated in *atmorc4/7* (see below) and is up-regulated only in *atmorc4/7*. On the other hand, the gene *SDC* [37] was much more highly up-regulated in *atmorc6* than it was in *atmorc4/7*, consistent with the use of its promoter in the forward genetic screen that resulted in isolation of *atmorc6* [25]. A similar plot comparing *atmorc4/6/7* versus *atmorc1/2/4/5/6/7* showed an extremely close correlation (Fig 3D and see S3 Fig). This demonstrates that AtMORC1, AtMORC2, and AtMORC5 [NP_196817; At5G13130; CRH5] do not have a significant impact on the transcriptome, consistent with the previous report indicating that *atmorc1/2* is equivalent to that of *atmorc6* and that the expression of *AtMORC5* is pollen specific [35].

### AtMORC4 and AtMORC7 play a role in plant defense

**Fig 4:**
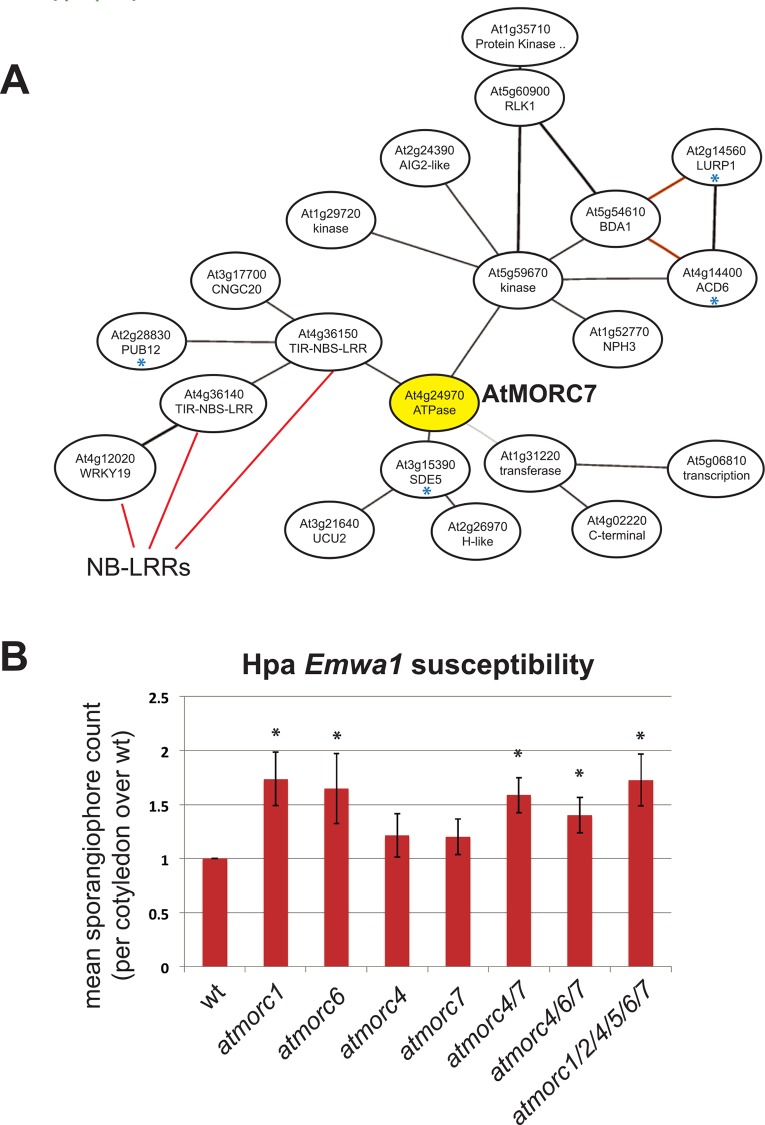
AtMORC4 and AtMORC7 act redundantly in pathogen defense. **(A)** ATTED-II microarray co-expression network for *AtMORC7* [http://atted.jp]. *AtMORC7*, shown in yellow, is co-expressed with multiple immunity related genes. Blue asterisk indicates genes with established roles in pathogen defense [39–42] and NB-LRRs are classic resistance genes [43]. **(B)** Mean Emwa1 *Hpa* sporangiophore count per cotyledon over wt, (4–5 days post inoculation of 10 day old seedlings, >100 cotyledons scored per genotype). Data from three individual replicates of the experiment. Error bars represent SEM. * indicates significant difference from wt (p-value < 0.05).

We performed GO term analysis on the genes misregulated in *atmorc4/7*, which revealed a striking enrichment for immune response genes, especially ‘response to chitin’ (p value = 2.3e^-47^) (S4 Fig). Interestingly, we had previously noted ‘response to chitin’, albeit with lower significance, (p < 6e^-4^), for genes misregulated in *atmorc6* [35]. Chitin is a component of the fungal cell wall and acts as a basal defense response elicitor [38]. In addition, *AtMORC7* appears in an RNA co-expression network with multiple disease resistance genes, including LURP1[39], PUB12[40], ACD6[41], SDE5[42] and three NB-LRR type proteins [43] (Fig 4A).

Since *LURP1* mutants are compromised in defense against the Emwa1 isolate of the oomycete pathogen, *Hyaloperonospora arabidopsidis (Hpa)* [39] and *atmorc1* was also identified as showing enhanced susceptibility to this pathogen [44], we challenged *atmorc1*, *atmorc6*, *atmorc4*, *atmorc7*, *atmorc4/7*, *atmorc4/6/7* and *atmorc1/2/4/5/6/7* with Emwa1 *Hpa*. We observed significantly increased susceptibility in *atmorc1*, *atmorc6*, *atmorc4/7*, *atmorc4/6/7* and *atmorc1/2/4/5/6/7* as compared to wt (Fig 4B). The individual *atmorc4* and *atmorc7* mutants did not show a difference from wild type, providing further support for the functional redundancy between AtMORC4 and AtMORC7. As we did not observe an additive increase in susceptibility in the higher order *atmorc* mutants, we reasoned that this might reflect non-additive changes in the transcriptome. Indeed, the *atmorc4/6/7* and *atmorc1/2/4/5/6/7* plants showed no further increase in expression of the ‘response to chitin’ (GO:0010200) gene set than did *atmorc4/7* (S5 Fig). While the mis-expression of specific genes in this set may contribute to pathogen susceptibility, it also remains possible that AtMORC proteins play a more direct role in defense [31,36,45]. Together, these results suggest that—in addition to AtMORC1—AtMORC6, AtMORC4, and AtMORC7 act as positive regulators of defense in *A*. *thaliana* against the oomycete *Hpa*.

### Chromocenter adjacent enrichment of AtMORC4 and AtMORC7 in the nucleus

**Fig 5:**
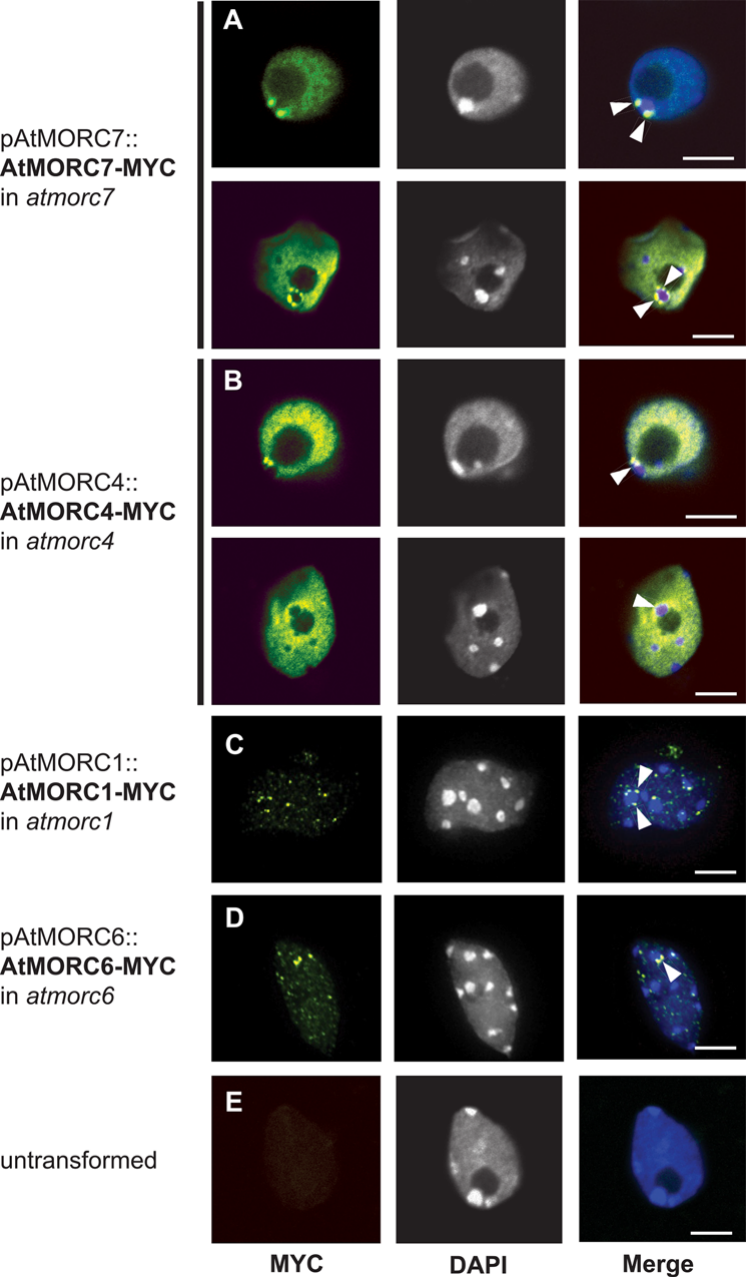
Chromocenter adjacent enrichment of AtMORC4 and AtMORC7 in the nucleus. **(A-D)** Representative examples of body forming AtMORC7-MYC, AtMORC4-MYC, At-MORC1-MYC, and AtMORC6-MYC nuclei, respectively. **(E)** Untransformed wt nucleus subjected to the same antibody staining and imaging procedure. Left panels = anti-MYC channel; middle panels = DAPI channel (gray scaled). DAPI stains DNA, defining the position of dense chromocenters as high intensity white foci; right panels = merged channels (DAPI in blue, MYC in green). White triangles indicate examples of chromocenter adjacent AtMORC localization. Scale bars = 5 μM.

In *Arabidopsis*, interphase chromosomes are organized into distinct chromosomal territories, with euchromatic arms looping out from condensed heterochromatic chromocenters [46–48]. These chromocenters constitute repeat and transposon-rich pericentromeric heterochromatin and are readily visible by light microscopy as intensely DAPI stained nuclear foci. AtMORC1 and AtMORC6 form punctate bodies adjacent to chromocenters and in *atmorc6* mutants, pericentromeric regions are decondensed, suggesting that AtMORC6 plays a role in higher order chromatin compaction at the interface of these transposon-rich regions [25,48]. Because AtMORC4 and AtMORC7 were found to target both genes and transposons, we determined their localization in the nucleus. Using pAtMORC4::AtMORC4-MYC and pAtMORC7::AtMORC7-MYC lines, we observed chromocenter adjacent bodies formed by both AtMORC4 and AtMORC7 (Fig 5A and 5B and S1 and S2 Videos). AtMORC7 bodies were generally more intensely stained than AtMORC4 bodies. Consistent with the effects of *atmorc4/7* mutation on euchromatic gene expression, AtMORC4 and AtMORC7 were also uniformly distributed throughout the nucleoplasm whereas AtMORC1 and AtMORC6 tended to appear as punctate nuclear foci (see Fig 5C and 5D and previously observed [25]). AtMORC4 and AtMORC7 staining was specifically excluded from chromocenters, but was frequently enriched along chromocenter boundaries, forming multiple foci or forming rings around chromocenters (Fig 5). The function of these nuclear bodies is currently unknown.

### The contribution of MORC to DNA methylation patterning

We utilized the *atmorc1/2/4/5/6/7* hextuple mutant to determine the contribution of AtMORCs to DNA methylation patterning. We performed whole-genome bisulfite sequencing (BS-seq), to examine DNA methylation at single cytosine resolution, in *atmorc1/2/4/5/6/7* as well as *atmorc4/7* and wt (2 biological replicates each). We also included the previously published BS-seq dataset for *atmorc6* [35] in our analysis. Global levels of methylation over the chromosomes were unaltered in any *AtMORC* knockout background in all three sequence-contexts (S6A Fig). Focusing specifically on loci that were de-repressed in *atmorc1/2/4/5/6/7*, we observed very little overall change in methylation upstream, downstream or throughout the gene body at these loci (S6B Fig). These results suggest that the most significant changes in transcription resulting from the loss of AtMORCs are not generally accompanied by losses in DNA methylation.

Next we examined the potential contribution of AtMORC to the different DNA methylation pathways. MET1 maintains CG methylation throughout the genome, CMT3 maintains the majority of CHG methylation, DRM2 maintains CHH methylation at RdDM sites, and CMT2 maintains CHH methylation in pericentromeric heterochromatin [3,7,8,10]. Using previously defined loci whose methylation is dependent upon these methyltransferases [8,49], we examined methylation levels in the *AtMORC* mutants. Again we found essentially no reduction in methylation in the AtMORC knockouts, suggesting that AtMORCs do not play a significant role in any of the major DNA methylation pathways in Arabidopsis (S7A Fig). We also tested whether AtMORCs might act downstream of DNA methylation from any of these specific methyltransferase pathways by plotting RNA-seq reads over differentially methylated regions (DMRs) defined as changing in the different methyltransferase mutant backgrounds; however, we did not observe any consistent changes in bulk levels of RNA in the *AtMORC* knockouts at these collections of methylated loci (S7B Fig).

**Fig 6:**
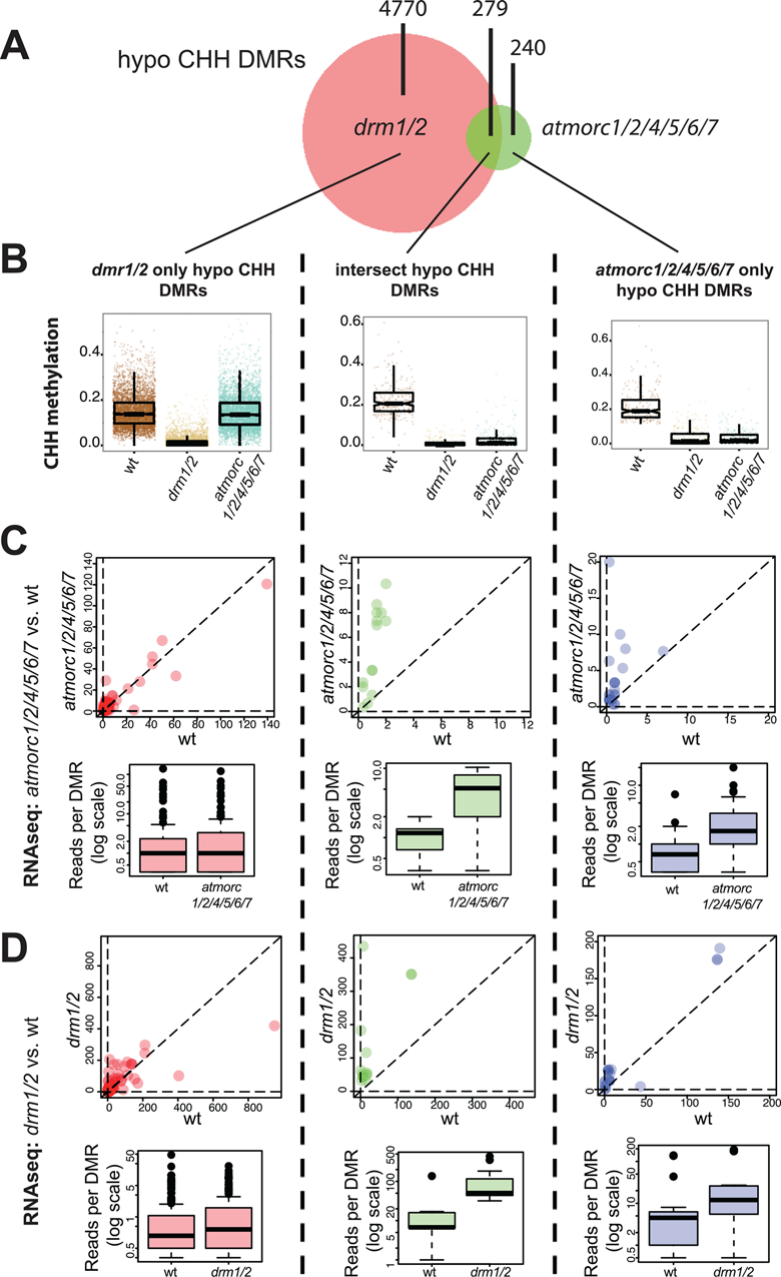
*atmorc* but not *drm1/2* specific hypomethylated CHH DMRs are associated with transcriptional de-repression. **(A)** Overlap between *atmorc1/2/4/5/6/7* hypo-CHH DMRs and *drm1/2* hypo-CHH DMRs. **(B)** Boxplot for CHH methylation levels in wt, *drm1/2*, and *atmorc1/2/4/5/6/7* at the hypo CHH DMR regions indicated. Note that although 241 loci were defined as *‘atmorc1/2/4/5/6/7* only’ in (**A**), they still lose significant of CHH methylation in in *drm1/2*, indicating that these regions are still likely targets of RdDM. **(C)** Upper: Scatter plot showing RNA-seq reads over DMR regions indicated from *atmorc1/2/4/5/6/7* vs. wt (average from three replicates each). Each dot represents a single DMR. Lower: Boxplots using the same RNA-seq data as above. **(D)** Same as in **(C)** except using RNA-seq data from *drm1/2* vs. wt from (data from GEO:GSE51304) [8] (average from two replicates each). In **(C)** and **(D)** only DMRs with transcripts detectable in both genotypes were included.

Since AtMORC6 has been implicated in transcriptional silencing at RdDM loci, reportedly interacting with members of the RdDM pathway [19,26], we examined whether there might be more localized changes in DNA methylation by parsing the genome into 100bp windows and searching for DMRs. We found 519 *atmorc1/2/4/5/6/7* hypomethylated CHH DMRs, 54% of which overlapped with *drm1/2* hypomethylated CHH DMRs (Fig 6A, S8A Fig). In addition, the remaining 46% of hypomethylated CHH DMRs that were called as being specific to *atmorc1/2/4/5/6/7* in fact showed dramatically reduced methylation in *drm1/2* (Fig 6B, right panel), suggesting that even though these DMRs did not make the stringent cutoff required to be a DMR, the majority of *atmorc1/2/4/5/6/7* hypomethylated DMRs correspond to sites of RNA directed DNA methylation. In contrast, only 2% of *atmorc1/2/4/5/6/7* hypomethylated DMRs exclusively overlapped with *cmt2* hypomethylated CHH DMRs (S8A Fig). We also checked whether these *atmorc1/2/4/5/6/7* hypomethylated CHH DMRs might be the result of spontaneous epi-allelic variation by comparison with a previously defined set of DMRs that are known to change states in the wild type [50], but found only a 3% overlap (S8B Fig). Together, these data suggest that AtMORCs are required for CHH methylation at a small subset of *drm1/2*-RdDM loci.

Comparing *atmorc6* with *atmorc4/7* at *atmorc1/2/4/5/6/7* hypo CHH DMRs, we found that *atmorc6* more strongly resembles that of *atmorc1/2/4/5/6/7* (S9 Fig). Interestingly, *atmorc4/7* and *atmorc6* do not appear to affect mutually exclusive regions, suggesting that AtMORC4/7 and AtMORC6 are required at overlapping target loci (S9A Fig). However, *atmorc4/7* generally showed less severe CHH methylation loss than *atmorc6* (S9A and S9B Fig), which is consistent with AtMORC4 and AtMORC7 being primarily involved in repression of protein-coding genes, and AtMORC6 being predominantly involved in repression of methylated elements.

Since the AtMORCs appear to be transcriptional repressors, we plotted RNA-seq data over the *atmorc1/2/4/5/6/7* hypomethylated CHH DMRs. We observed a clear increase in bulk levels of RNA over these sites in the *atmorc1/2/4/5/6/7* knockout (S10A Fig). While this result might seem intuitive, this was not the case for *drm1/2* hypomethylated CHH DMRs, where loss of *DRM1/2* did not result in significant transcriptional re-activation (S10B Fig and [8]). To determine whether the overall change in transcription seen in *atmorc1/2/4/5/6/7* knockout is caused by a small number of jackpot sites or is the result of many DMRs becoming transcriptionally reactivated at a moderate level, we plotted RNA-seq reads from individual DMRs (Fig 6C and 6D). We found that *atmorc1/2/4/5/6/7* hypomethylated CHH DMRs were frequently characterized by transcriptional de-repression, while *drm1/2* exclusive hypomethylated CHH sites were not. Interestingly, the *atmorc1/2/4/5/6/7* defined hypomethylated CHH sites were also transcriptionally reactivated in the *drm1/2* background (Fig 6D). Thus this set of sites is susceptible to transcriptional depression when CHH methylation is lost, either by loss of RdDM or by loss of MORC function.

**Fig 7:**
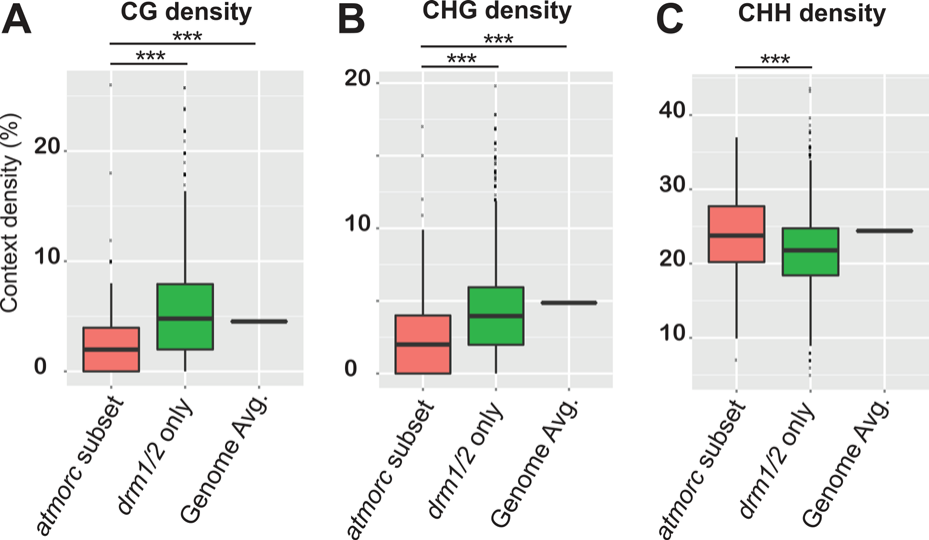
*atmorc* defined transcriptionally activatable subset of RdDM loci are characterized by reduced symmetric CG and CHG site density. In Fig 6 we showed that *atmorc* hypo CHH DMRs defined a subset of RdDM loci that become transcriptionally reactivated when CHH methylation is lost. Here we calculated density per base pair of CG **(A)**, CHG **(B),** and CHH **(C)** sites at this subset of RdDM loci, termed ‘*atmorc* subset’ (defined as the intersect between *atmorc1/2/4/5/6/7* and *drm1/2* hypo CHH DMRs, n = 279, see Fig 6A), and compare it to the rest of RdDM loci, termed ‘drm1/2 only’ (n = 4770, see Fig 6A), and the genome average ‘Genome Avg.’. While asymmetric CHH density is relatively high at the ‘*atmorc* subset’, the density of sites for symmetric CG and CHG methylation are depleted by approximately half as compared to the ‘*drm1/2* only’ loci and the genome average. Counts of CG, CHG, and CHH reflect presence on either strand, ie 2% CG indicates two CpG sites—one on each strand—for every 100bps. *** indicates statistically significant difference, p<0.001.

In order to determine if the 519 *atmorc1/2/4/5/6/7* hypomethylated DMR regions might have unique qualities that distinguish them from other sites that do not lose CHH methylation, we analyzed their DNA sequence composition. Interestingly, when we calculated CG, CHG, and CHH density, we found that the *atmorc1/2/4/5/6/7* defined subset had significantly fewer CG and CHG sites as compared to the rest of the RdDM loci and compared to the genome average (Fig 7). An attractive hypothesis therefore is that a low density of symmetric methylation (due to a low density of methylatable sites) may not be sufficient to maintain silencing once asymmetric CHH methylation is lost, which would explain why these particular regions become reactivated in *drm1/2*. Since AtMORCs are not generally required for CHH methylation maintenance, it would then seem likely that AtMORCs primary role would be to help maintain transcriptional repression at these regions of diffuse symmetric methylation and poised transcriptional potential. The transcriptional reactivation of these sites in *atmorc* may then secondarily lead to loss of CHH methylation at these loci, and it is indeed known that positive epigenetic marks associated with transcription can lead to a loss of RdDM function [14,51,52]. In addition, symmetric CG methylation plays a role in the stable association of Pol V to chromatin, and thus perpetuates RdDM and CHH methylation [18]. Thus we hypothesize that this unique set of 519 *atmorc1/2/4/5/6/7* hypomethylated DMR regions experience a loss of methylation because they are both depleted in symmetric methylation and because they become transcriptionally reactivated in *atmorc* mutants.

### Conclusion

In this study, we established a role for the previously uncharacterized *AtMORC4* and *AtMORC7* genes in widespread repression of protein-coding genes and in pathogen defense. We found that these proteins act partially redundantly, forming mututally exclusive homomeric complexes, which explains why they have not previously been identified in forward genetic screens. In addition, AtMORC4 and AtMORC7 formed bodies adjacent to chromocenters while also showing localization throughout the nucleoplasm. By analysing a compound mutant devoid of all MORC function, we showed that AtMORC is not a key component in the maintenance of any of the major DNA methylation pathways and that major changes in transcription were not generally accompanied by loss of DNA methyation. However, at a small subset of RdDM targets (approximately 5%), AtMORC was required for both methylation and silencing, suggesting that these methylation losses are likely an indirect consequence of the loss of gene silencing. These findings reconcile our laboratory’s previous reports of methylation-independent silencing [25] with that of other laboratories reporting hypomethylation at specific de-repressed reporter loci in *atmorc6* mutant backgrounds [26,27].

We recently reported that mouse MORC1 is required for DNA methylation and silencing at a specific subset of transposon promoters that are normally methylated at a developmentally late stage during the wave of global *de novo* methylation in the male germ line [53]. As in Arabidopsis, there were no genome wide changes in DNA methylation in the mouse *morc1* mutant, but specific methylation defects at a class of transposons that failed to establish silencing. These commonalities suggest that *Arabidopsis* MORCs may act similarly to mammalian MORC1, to maintain silencing at loci that are poised for transcriptional de-repression, with DNA hypomethylation as a secondary effect.

Nuclear localization of AtMORC4 and AtMORC7 broadly reflected that of their euchromatic gene and pericentromeric transposon targets, with both chromocenter adjacent enrichment and distribution throughout the nucleus. Since we previously reported that AtMORC6 and AtMORC1 form chromocenter adjacent bodies [25] (and see Fig 5), this appears to be a general feature of *Arabidopsis* MORC proteins, although the function of these bodies is at present completely unknown. In the future, it will be important to determine the precise molecular mechanisms by which MORC proteins interact with chromatin and regulate gene expression.

## Materials and Methods

### Plant materials and growth

Wild-type and all mutant lines are from the ecotype Columbia (Col-0) and were grown under either continuous light (S1 Fig, Fig 2) or long days (16 hour light—all other experiment). The T-DNA lines used in this study were: *atmorc1-2* (gene AT4G36290) SAIL_893_B06 (aka *crt1-2*), *atmorc2-1* (gene AT4G36280) SALK_072774C (aka *crh1-1*), *atmorc3-2* (gene AT4G36270) SALK_043244, *atmorc4-1* (gene AT5G50780) GK-249F08 (aka *crh4-2*), *atmorc5-1* (gene AT5G13130) SALK_049050C (aka *crh5-2*), *atmorc6-3* (gene AT1G19100) GABI_599B06 (aka *crh6-5*), and *atmorc7-1* (gene AT4G24970) SALK_051729 (aka *crh3-1*). T-DNAs were confirmed by PCR based genotyping. Primer sequences are described in S1 Table.

### Plasmid construction and transgenic plants

The pAtMORC4::AtMORC4-MYC, pAtMORC4::AtMORC4-FLAG, pAtMORC7::AtMORC7-MYC, and pAtMORC7::AtMORC7-MYC constructs were generated by the same method described in [35]. Briefly, the AtMORC4 and AtMORC7 genomic regions, including ~1 kb upstream from the transcriptional start sites, were PCR amplified and cloned into a pENTR/D-TOPO vector (#K2400-20, Thermo Fisher). The cloned genomic regions were then transferred into a pEG302 based binary destination vector that included a MYC or FLAG epitope tag at the C-terminus via a Gateway LR Clonase II reaction (#11791–100, Thermo Fisher). *Agrobacterium tumfaciens* AGLO strain carrying these constructs were used to transform *A*. *thaliana* plants in their respective mutant backgrounds using the floral dip method [54].

### BS-seq libraries

2–3 leaves from individual 3-week old plants were used to make individual BS-seq libraries based on methods described by [49]. Briefly, genomic DNA was extracted using DNeasy Plant Mini kit (#69106) and 500ng was sheared using the Covaris S2 instrument. Libraries were generated using the Kapa Hyper Prep Kit (#KK8502) with bisulfite conversion using the EZ DNA Methylation Lightning Kit (#D5030). Libraries were sequenced on a HiSeq 2000 (Illumina).

### RNA-seq libraries and RT-PCRs

RNA was extracted from 2–3 leaves of 3-week old plants using Trizol reagent and DNAse treated using TURBO DNA-free kit (#AM1907). For RNA-seq, 1–2.5 μg of RNA starting material per library was first rRNA depleted using Epicentre RiboZero (#MRZPL1224) prior to library generation using Epicentre ScriptSeqv2 (#SSV21124). Libraries were sequenced on a HiSeq 2000 (Illumina). For RT-PCRs, cDNA was generated using SuperScript III (#18080–044, ThermoFisher) with random hexamer priming. The samples were digested with RNAse H in accordance with manufacturer’s protocol. RT–PCR was then performed with iQ SYBR Green Mastermix (BioRad) using an Agilent Technologies Mx3005p qPCR System (Stratagene).

### *Hpa* assay

*Hyaloperonospora arabidopsidis* (Hpa) isolate Emwa1 was propagated on the susceptible *Arabidopsis* ecotype Ws. Conidiospores of Hpa strain Emwa1 were resuspended in autoclaved RO-water at a concentration of 3×10^4^ spores/mL and spray-inoculated onto 10-day old seedlings. Inoculated plants were covered with a lid to increase humidity and grown at 19°C under a 9-hour light period. Sporangiophores per cotyledon were counted 4 to 5 days post inoculation using a Leica M205 FA stereoscope. The experiments were repeated 3 times and the sporangiophores on approximately 100 cotyledons per genotype were counted in each experiment.

### Co-Immunoprecipitation (Co-IP) and Immunoprecipitation Mass spectrometry (IP-MS)

Co-IP and IP-MS on pAtMORC4::AtMORC4-MYC/FLAG and pAtMORC7::AtMORC7-MYC/FLAG lines were performed as previously described [35]. For IP-MS, M2 magnetic FLAG-beads (SIGMA, M8823) were added to the supernatant and immunoprecipitated proteins were eluted using 3×FLAG peptides (SIGMA, F4799). The MS was performed as described by [55]. For the Co-IPs, we added 100 μL M2 magnetic FLAG-beads (SIGMA, M8823) to the supernatant for pulldown. For the western blots, we used HRP-coupled FLAG-specific antibody (SIGMA, A8592) and MYC-specific antibodies (Pierce, MA1-980).

### Nuclear immunofluorescence

Nuclear immunofluorescence experiments for AtMORC4/7-MYC tagged lines were performed based on the method described in [25]. Leaves from three-week old plants were fixed in 4% paraformaldehyde in TRIS buffer (10 mM TRIS pH 7.5, 10 mM EDTA, and 100 mM NaCl) for 20 minutes and washed twice in TRIS buffer. Leaves were chopped in 200–400 microliters lysis buffer (15 mM TRIS pH 7.5, 2 mM EDTA, 0.5 mM spermine, 80 mM KCl, 20 mM NaCl, and 0.1% Triton X-100) and filtered through a 3 μM cell strainer (Corning, #352235). 5 μL of nuclei suspension was added to 12 μL of sorting buffer (100mM TRIS pH 7.5, 50mM KCl, 2mM MgCl2, 0.05% Tween-20, and 20.5% sucrose) and air dried on chloroform dipped microscope slides for two hours and then post-fixed in 4% paraformaldehyde in PBS for 20 minutes. Slides were washed three times in PBS and incubated in blocking buffer (3% BSA, and 10% horse serum in PBS) for 30 minutes at 37°C. Nuclei were incubated at 4°C overnight in mouse monoclonal antibody against c-Myc (9E10, Abcam ab32; 1:200). Slides were washed in PBS and incubated with goat anti-mouse FITC antibody (Abcam, ab7064; 1:200) for 90 minutes at room temperature. Following PBS washes, nuclei were counterstained and mounted in Vectashield mounting media with DAPI (Vector, H-1200). Nuclei were analyzed with a Zeiss LSM 710 Confocal microscope at 63X or 100X magnification using Zen software.

### Bioinformatics

For RNA-seq analysis, reads were aligned with TopHat, including the fr-secondstrand parameter. Cufflinks was used to generate count data using annotation from TAIR10 that was fed into the DEseq2 package in R for differential expression analysis. For BS-seq, reads were aligned using BSMAP with methylation levels calculated and DMRs defined as previously described [49]. For the *atmorc* DMRs, each biological replicated (two per mutant) was compared against two wild type biological replicates from the same experiment, requiring that the DMR be identified in all four mutant vs. wt comparisons to be considered a ‘true’ DMR. The *dmr1/2*, *cmt2*, *cmt3*, and *met1* DMRs were previously defined [49], using a single mutant biological replicate compared against three biological wild type replicates.

### Data deposition

The data reported in this paper have been deposited in the Gene Expression Omnibus (GEO) database (accession number GSE78836).

## Supporting Information

S1 Fig *atmorc4/7* double mutant shows de-repression at AtMORC6 transposon targets. **(A)** RT-PCR on cDNA derived from *atmorc4-1/atmorc7-1* double mutant compared to wt showing no detectable wild type transcript in these T-DNA mutants. Primers were designed to span the T-DNA region in *atmorc4-1* (upper) and *atmorc7-1* (middle) (S1 Table). UBQ10 (lower) was amplified as a loading control (S1 Table). **(B)** RT-PCR at AtMORC6 targets indicated using the genotypes indicated. Error bars indicate standard error of the mean (SEM).
(PDF)

S2 Fig *AtMORC3* is likely to be a pseudogene. **(A)** TAIR predicted gene structure for *AtMORC1*, *AtMORC2*, and *AtMORC3*. Boxes = exons, light blue = UTR, and dark blue = CDS. *AtMORC1*, *AtMORC2*, and *AtMORC3* are highly related to one another, (see Fig 1A, and **(B)** below), encode the same number of exons, and lie directly adjacent to one another on *A*. *thaliana* chromosome four, indicating that they likely arose from a tandem duplication event. In the predicted 5’ UTR of *AtMORC3*, there is an ATG start codon. However, a G to A mutation causes a W to Stop codon in exon three. BLAST of this *in silico* translated region identifies all other AtMORC proteins. However, because this ORF is predicted to be too small, TAIR finds the next in-frame ATG in exon 5, annotating this to be the translational start. If this protein were made, it would be N-terminally truncated, missing half of the GHKL ATPase including two out of the four motifs thought to be essential for ATP binding [28,29]. **(B)** Phylogenetic reconstruction of *AtMORC* genes in *Arabidopsis thaliana* and close relatives, *Capsella rubella* and *Arabidopsis lyrata*. The tandem arrangement of *AtMORC1*, *AtMORC2*, and *AtMORC3*, and the premature stop codon identified in *AtMORC3* is consistent with the pseudogenisation of a redundant paralogue. Therefore, we checked whether *AtMORC1*, *AtMORC2*, and *AtMORC3* are also present in *A*. *thaliana* sister species. We found that while the closely related *A*. *lyrata* encodes a single copy of each of *A*. *thaliana*’s *AtMORC* genes, the slightly more distantly related *C*. *rubella* does not encode a copy of either *AtMORC2* or *AtMORC3* (and encodes two copies of *AtMORC4*). Therefore *C*. *rubella* has either lost its versions of *AtMORC2*/*AtMORC3* or the tandem duplication of *AtMORC1* occurred after the divergence of *A*. *thaliana* and *A*. *lyrata* from *C*. *rubella*. In either scenario, it suggests that *AtMORC2* and *AtMORC3* are likely non-essential and may act redundantly with *AtMORC1*. In support of this hypothesis, we have already shown that *AtMORC2* is redundant with *AtMORC1* [35]. **(C)** Positions of the SALK_000009 and SALK_043244 insertions in *AtMORC3*. **(D)** Sequence of SALK_043244 T-DNA homozygous insert in *AtMORC3*. As the SALK_000009 line, which has a T-DNA insert in the 5’ UTR of *AtMORC3*, was found to be embryonic lethal [36], we took an independent *AtMORC3* T-DNA line to homozygosity and sequence confirmed the presence of the insert in exon 11, finding that this line displays no discernable phenotype. Together with the premature stop codon in exon 3, it is likely that *AtMORC3* is a non-functional pseudogene in Columbia-0.
(PDF)

S3 Fig Comparison of RNA-seq in *atmorc4/6/7* vs. *atmorc1/2/4/5/6/7*. **(A)** Overlap between *atmorc4/6/7* and *atmorc1/2/4/5/6/7* upregulated DEGs. **(B)** Boxplot showing the FPKM (fragments per kilobase per million reads) for the 241 genes in *atmorc1/2/4/5/6/7* that did not overlap with *atmorc4/6/7* (purple section in (**A**)). This shows that while these genes did not make the significance cutoff required to be called DEGs in *atmorc4/6/7*, they still show the same trend for upregulation, indicating that the addition of *atmorc1*, *2* and *5* has very little additional impact on the transcriptome (also see Fig 3D).
(PDF)

S4 Fig DEGs in *atmorc4/7* are highly enriched for pathogen defense. **(A)** Top ten listed GO term categories from *atmorc4/7* misregulated genes (FDR<0.05) [http://bioinfo.cau.edu.cn/agriGO] identified RNA-seq round 2 (see Fig 3). **(B)** Top ten listed GO term categories from *atmorc4/7* misregulated genes (FDR<0.05) [http://bioinfo.cau.edu.cn/agriGO] identified RNA-seq round 1 (see Fig 1).
(PDF)

S5 Fig No additive transcriptional effect at ‘response to chitin’ genes in higher-order *atmorc* knockouts. Boxplot showing FPKMs at the ‘response to chitin’ gene set (GO:0010200) in the genotypes indicated.
(PDF)

S6 Fig Negligible DNA methylation changes genome wide and at AtMORC targets in *AtMORC* knockouts. **(A)** Genome wide profiles of CG, CHG, and CHH context methylation in the wt, *atmorc4/7*, *atmorc6*, and *atmorc1/2/4/5/6/7* backgrounds. Average of two biological replicates of each genotype, except *atmorc6* (data obtained from GSE54677) [35]. **(B)** Metaplot of methylation levels in wt, *atmorc4/7* and *atmorc1/2/4/5/6/7* over DEGs (>2 fold change, FDR<0.05) in *atmorc1/2/4/5/6/7* background, in CG, CHG and CHH contexts. TSS = transcriptional start site, TTS = transcriptional termination site.
(PDF)

S7 Fig Loss of AtMORC does not significantly impact any of the major DNA methylation pathways and does not act downstream of DNA methylation. **(A)** Boxplots for methylation levels at *drm1/2* CHH, *cmt2* CHH, *cmt3* CHG, and *met1* CG defined hypomethylated DMRs [8,49] in the wt, *atmorc4/7*, *atmorc6*, *atmorc1/2/4/5/6/7*, and control methyltransferase mutant backgrounds indicated. **(B)** RNA-seq from wt and *atmorc1/2/4/5/6/7* (black and green, respectively, three replicates each, see Fig 3) over methylated loci defined by *drm1/2* CHH, *cmt2* CHH, *cmt3* CHG, and *met1* CG hypo DMRs (as in (**A**)).
(PDF)

S8 Fig *atmorc1/2/4/5/6/7* hypo CHH DMRs overlap with RdDM sites. **(A)** Overlap of *atmorc1/2/4/5/6/7* defined hypo CHH DMRs with previously defined *drm1/2* and *cmt2* hypo CHH DMRs [8,49]. **(B)** Overlap of *atmorc1/2/4/5/6/7* hypo CHH DMRs with CHH loci prone to spontaneous epiallelic variation [50].
(PDF)

S9 Fig Comparison of *atmorc6* with *atmorc4/7* at *atmorc1/2/4/5/6/7* hypo CHH DMRs. (A) Heatmap showing CHH methylation levels at all *atmorc1/2/4/5/6/7* hypo CHH DMRs in the genotypes indicated. *atmorc4/7* and *atmorc6* appear to affect many similar targets. Scale 0–0.6 indicates CHH methylation level. (B) Boxplot for methylation levels at same *atmorc1/2/4/5/6/7* hypo CHH DMRs as in (A). *drm1/2* is used as a control in (A) and (B), and demonstrates that *atmorc* hypo CHH DMRs are primarily RdDM target loci.
(PDF)

S10 Fig *atmorc1/2/4/5/6/7* hypo CHH DMRs show evidence for transcriptional de-repression. **(A)** RNA-seq metaplot of wt vs. *atmorc1/2/4/5/6/7* (black and green, respectively, three replicates each, see Fig 3) over *atmorc1/2/4/5/6/7* defined hypo CHH DMRs. **(B)** RNA-seq metaplot of wt vs. *drm1/2* (black and red, respectively, two replicates each) over drm1/2 hypo CHH DMRs (data from GEO:GSE51304) [8].
(PDF)

S1 Video AtMORC7-MYC rotate. z-stack at 0.83 μM intervals through the AtMORC7-MYC expressing nucleus depicted in Fig 5A was rendered in 3D with interpolation and rotated 360 degrees about the *y*-axis. Blue channel = DAPI staining; green channel = anti-MYC staining.
(AVI)

S2 Video AtMORC7-MYC stack. z-stack at 0.83 μM intervals through the AtMORC7-MYC expressing nucleus depicted in Fig 5A. z-stack slices from the furthest to closest depth are shown in sequence (5 frames per second), illustrating the presence of AtMORC7-MYC bodies first at one chromocenter (upper middle of nucleolus) and then more prominently at another (middle left, between nucleolus and nuclear periphery). Blue channel = DAPI staining; green channel = anti MYC staining. Scale bar = 2 μM
(AVI)

S1 Table Primers used in this study. List of relevant primers used in the study.
(PDF)
