# Supplementary figures and images for: Arabidopsis AtMORC4 and AtMORC7 Form Nuclear Bodies and Repress a Large Number of Protein-Coding Genes
Source: PLoS Genet. 2016 May 12;12(5):e1005998. doi: 10.1371/journal.pgen.1005998 (PMC4865129; doi:10.1371/journal.pgen.1005998)

**Fig. S1: *atmorc4/7* double mutant shows de-repression at AtMORC6 transposon targets.**

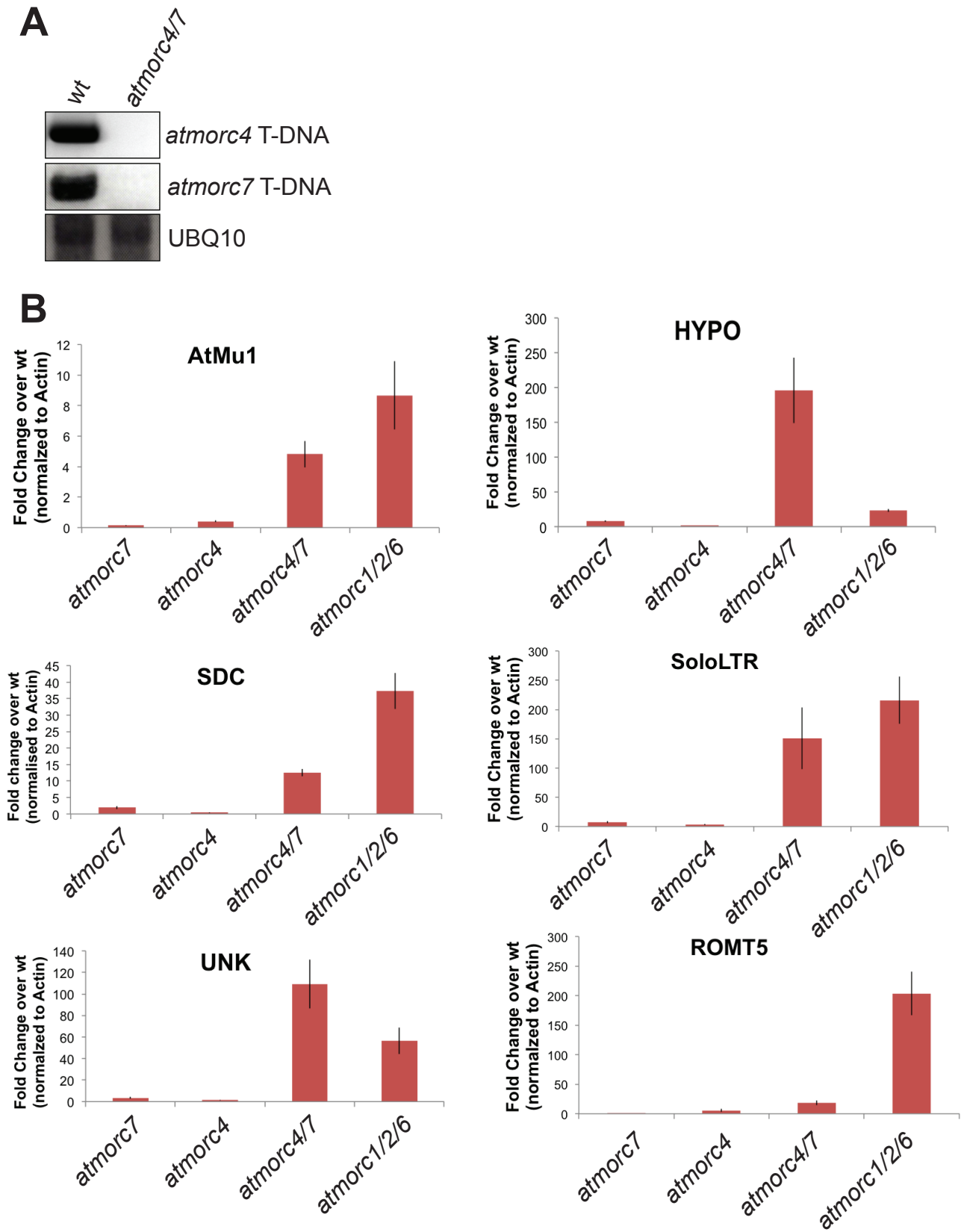

Supplement: S1 Fig — (A) RT-PCR on cDNA derived from atmorc4-1/atmorc7-1 double mutant compared to wt showing no detectable wild type transcript in these T-DNA mutants. Primers were designed to span the T-DNA region in atmorc4-1 (upper) and atmorc7-1 (middle) (S1 Table). UBQ10 (lower) was amplified as a loading control (S1 Table). (B) RT-PCR at AtMORC6 targets indicated using the genotypes indicated. Error bars indicate standard error of the mean (SEM). (PDF) [file pgen.1005998.s001.pdf]

**Fig. S2: *AtMORC3* is likely to be a pseudogene.**

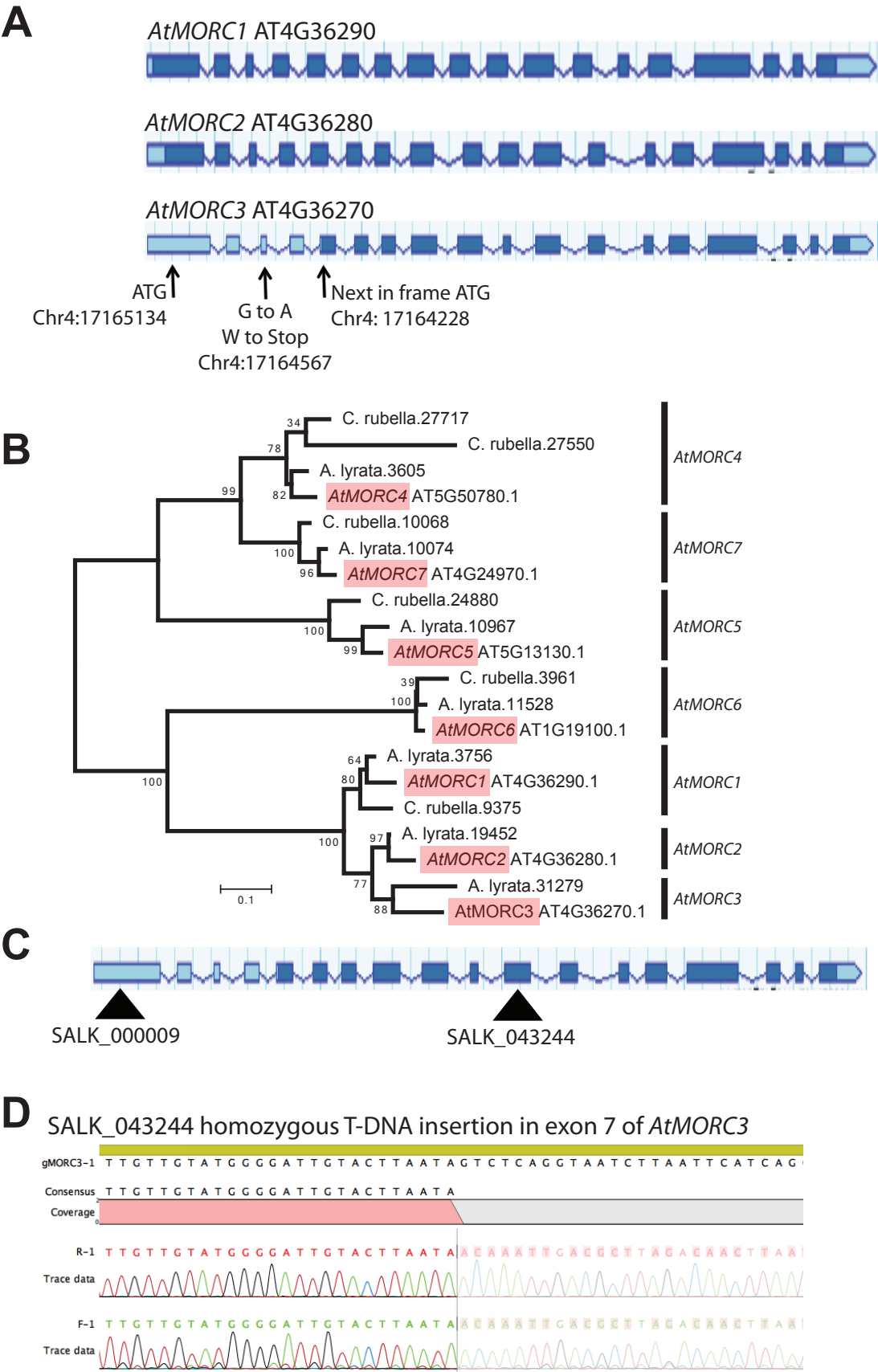

Supplement: S2 Fig — (A) TAIR predicted gene structure for AtMORC1, AtMORC2, and AtMORC3. Boxes = exons, light blue = UTR, and dark blue = CDS. AtMORC1, AtMORC2, and AtMORC3 are highly related to one another, (see Fig 1A, and (B) below), encode the same number of exons, and lie directly adjacent to one another on A. thaliana chromosome four, indicating that they likely arose from a tandem duplication event. In the predicted 5’ UTR of AtMORC3, there is an ATG start codon. However, a G to A mutation causes a W to Stop codon in exon three. BLAST of this in silico translated region identifies all other AtMORC proteins. However, because this ORF is predicted to be too small, TAIR finds the next in-frame ATG in exon 5, annotating this to be the translational start. If this protein were made, it would be N-terminally truncated, missing half of the GHKL ATPase including two out of the four motifs thought to be essential for ATP binding [28,29]. (B) Phylogenetic reconstruction of AtMORC genes in Arabidopsis thaliana and close relatives, Capsella rubella and Arabidopsis lyrata. The tandem arrangement of AtMORC1, AtMORC2, and AtMORC3, and the premature stop codon identified in AtMORC3 is consistent with the pseudogenisation of a redundant paralogue. Therefore, we checked whether AtMORC1, AtMORC2, and AtMORC3 are also present in A. thaliana sister species. We found that while the closely related A. lyrata encodes a single copy of each of A. thaliana’s AtMORC genes, the slightly more distantly related C. rubella does not encode a copy of either AtMORC2 or AtMORC3 (and encodes two copies of AtMORC4). Therefore C. rubella has either lost its versions of AtMORC2/AtMORC3 or the tandem duplication of AtMORC1 occurred after the divergence of A. thaliana and A. lyrata from C. rubella. In either scenario, it suggests that AtMORC2 and AtMORC3 are likely non-essential and may act redundantly with AtMORC1. In support of this hypothesis, we have already shown that AtMORC2 is redundant with AtMORC1 [35]. (C) Pos [file pgen.1005998.s002.pdf]

**Fig. S3: Comparison of RNA-seq in *atmorc4/6/7* vs. *atmorc1/2/4/5/6/7*.**

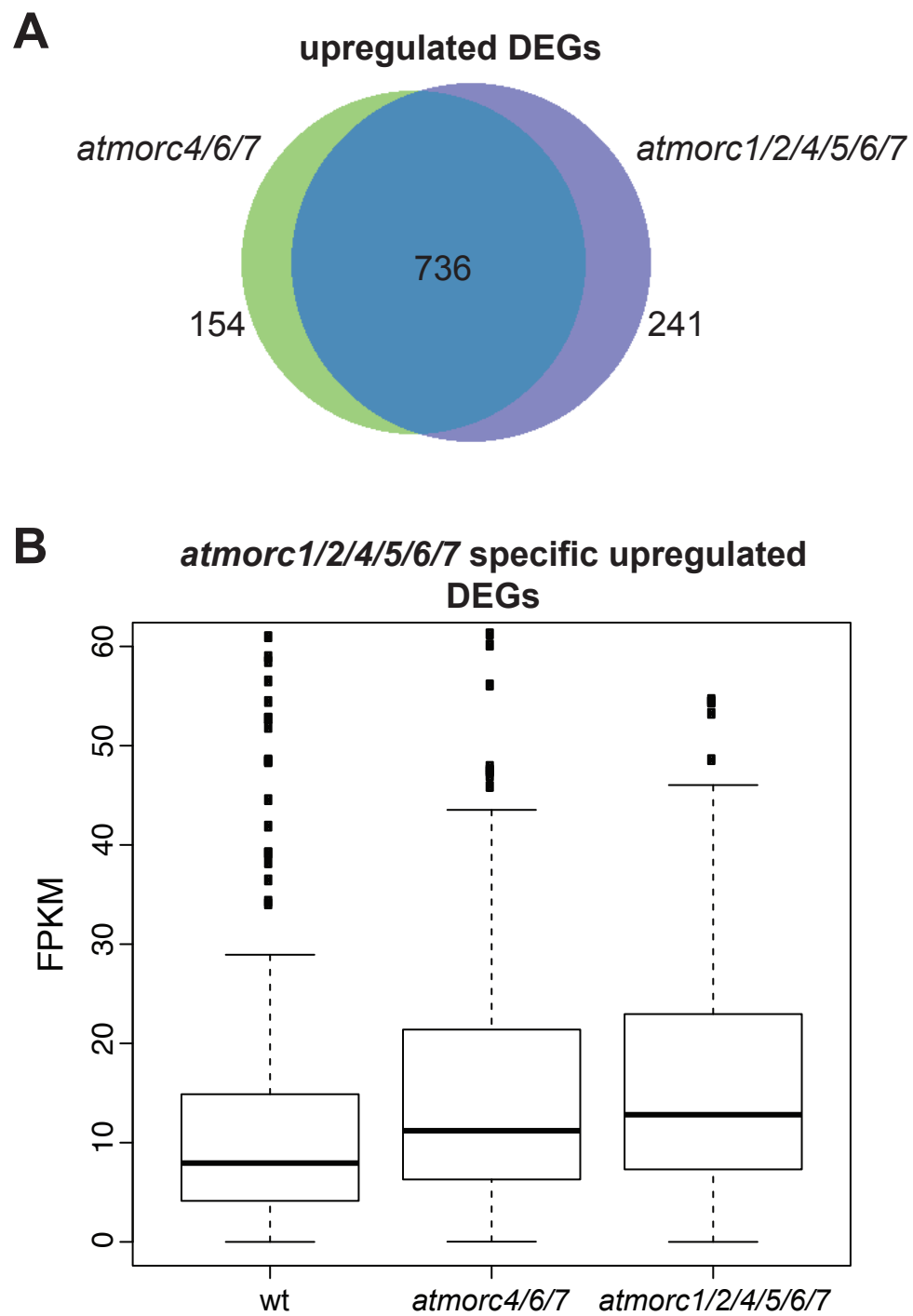

Supplement: S3 Fig — (A) Overlap between atmorc4/6/7 and atmorc1/2/4/5/6/7 upregulated DEGs. (B) Boxplot showing the FPKM (fragments per kilobase per million reads) for the 241 genes in atmorc1/2/4/5/6/7 that did not overlap with atmorc4/6/7 (purple section in (A)). This shows that while these genes did not make the significance cutoff required to be called DEGs in atmorc4/6/7, they still show the same trend for upregulation, indicating that the addition of atmorc1, 2 and 5 has very little additional impact on the transcriptome (also see Fig 3D). (PDF) [file pgen.1005998.s003.pdf]

**Fig. S9: Comparison of *atmorc6* with *atmorc4/7* at *atmorc1/2/4/5/6/7* hypo CHH DMRs.**

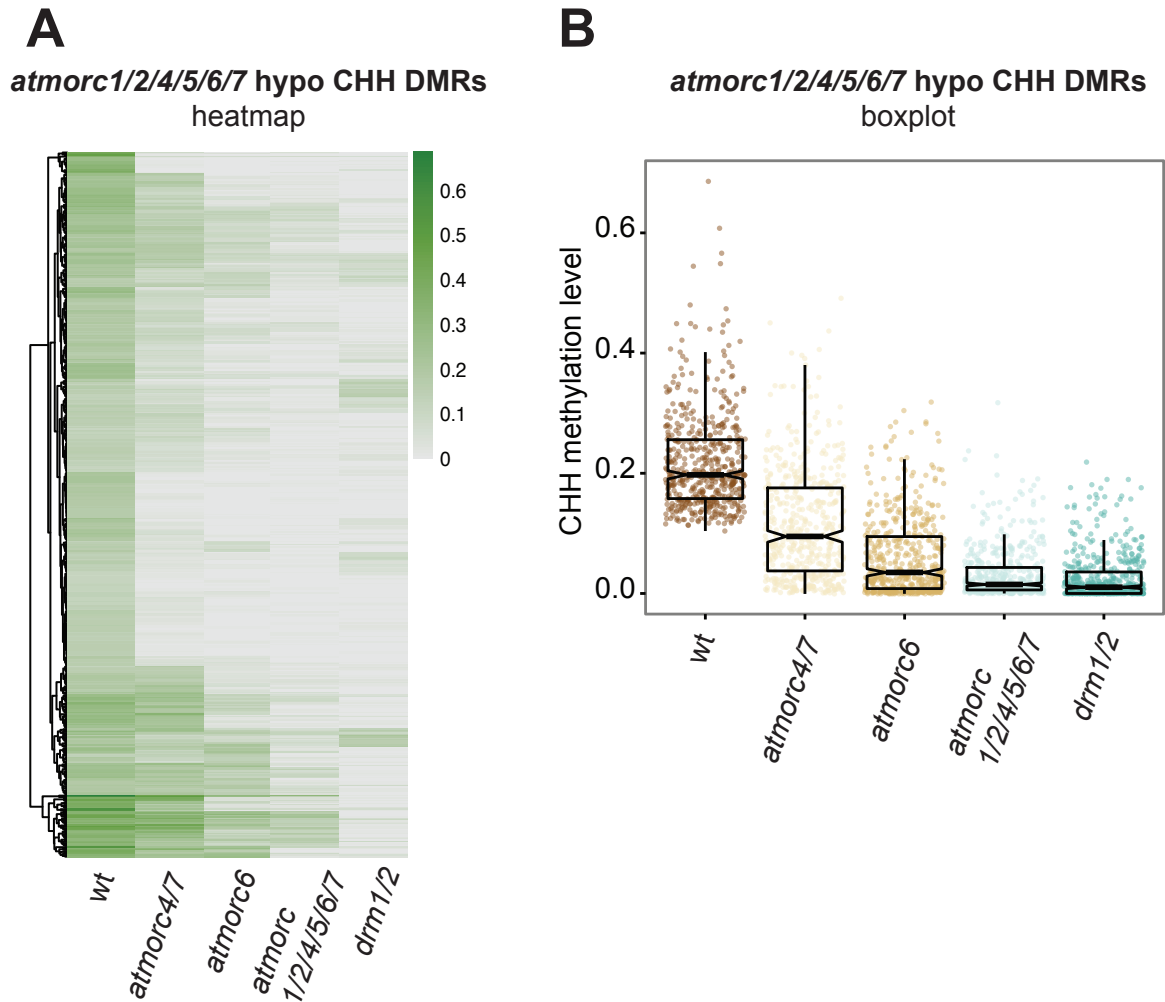

Supplement: S9 Fig — (A) Heatmap showing CHH methylation levels at all atmorc1/2/4/5/6/7 hypo CHH DMRs in the genotypes indicated. atmorc4/7 and atmorc6 appear to affect many similar targets. Scale 0–0.6 indicates CHH methylation level. (B) Boxplot for methylation levels at same atmorc1/2/4/5/6/7 hypo CHH DMRs as in (A). drm1/2 is used as a control in (A) and (B), and demonstrates that atmorc hypo CHH DMRs are primarily RdDM target loci. (PDF) [file pgen.1005998.s009.pdf]
