# Supplementary material for: Arabidopsis AtMORC4 and AtMORC7 Form Nuclear Bodies and Repress a Large Number of Protein-Coding Genes
Source: PLoS Genet. 2016 May 12;12(5):e1005998. doi: 10.1371/journal.pgen.1005998 (PMC4865129; doi:10.1371/journal.pgen.1005998)
Supplement: S4 Fig — (A) Top ten listed GO term categories from atmorc4/7 misregulated genes (FDR<0.05) [http://bioinfo.cau.edu.cn/agriGO] identified RNA-seq round 2 (see Fig 3). (B) Top ten listed GO term categories from atmorc4/7 misregulated genes (FDR<0.05) [http://bioinfo.cau.edu.cn/agriGO] identified RNA-seq round 1 (see Fig 1). (PDF) [file pgen.1005998.s004.pdf]

Fig. S4. DEGs in *atmorc4/7* are highly enriched for pathogen defense.

A

GO term	Ontology	Description	Number in input list	Number in BG/Ref	p-value	FDR
GO:0010200	P	response to chitin	57	151	4.2e-47	6.5e-44
GO:0009743	P	response to carbohydrate stimulus	63	240	5.8e-44	4.5e-41
GO:0050896	P	response to stimulus	229	4057	2.2e-43	1.1e-40
GO:0010033	P	response to organic substance	119	1342	5.1e-38	2e-35
GO:0042221	P	response to chemical stimulus	142	2085	1.1e-33	3.3e-31
GO:0006950	P	response to stress	138	2320	3.7e-27	9.5e-25
GO:0006952	P	defense response	66	766	1.2e-20	2.7e-18
GO:0002376	P	immune system process	43	368	3.6e-18	6.2e-16
GO:0006955	P	immune response	43	367	3.3e-18	6.2e-16
GO:0006468	P	protein amino acid phosphorylation	67	946	7.5e-17	1.2e-14

B

GO term	Ontology	Description	Number in input list	Number in BG/Ref	p-value	FDR
GO:0050896	P	response to stimulus	154	4057	1.1e-31	1.4e-28
GO:0042221	P	response to chemical stimulus	102	2085	2.4e-28	1.5e-25
GO:0010033	P	response to organic substance	79	1342	1.2e-26	5e-24
GO:0006950	P	response to stress	94	2320	1.5e-20	4.8e-18
GO:0009719	P	response to endogenous stimulus	61	1068	4.7e-20	1.2e-17
GO:0009725	P	response to hormone stimulus	55	982	8.3e-18	1.8e-15
GO:0010200	P	response to chitin	21	151	2.8e-14	5.2e-12
GO:0009743	P	response to carbohydrate stimulus	25	240	4.1e-14	6.6e-12
GO:0009611	P	response to wounding	21	197	3e-12	4.4e-10
GO:0009620	P	response to fungus	18	158	4e-11	5.2e-09
