# Supplementary material for: Arabidopsis AtMORC4 and AtMORC7 Form Nuclear Bodies and Repress a Large Number of Protein-Coding Genes
Source: PLoS Genet. 2016 May 12;12(5):e1005998. doi: 10.1371/journal.pgen.1005998 (PMC4865129; doi:10.1371/journal.pgen.1005998)

Fig. S5: No additive transcriptional effect at ‘response to chitin’ genes in higher-order *atmorc* knockouts.

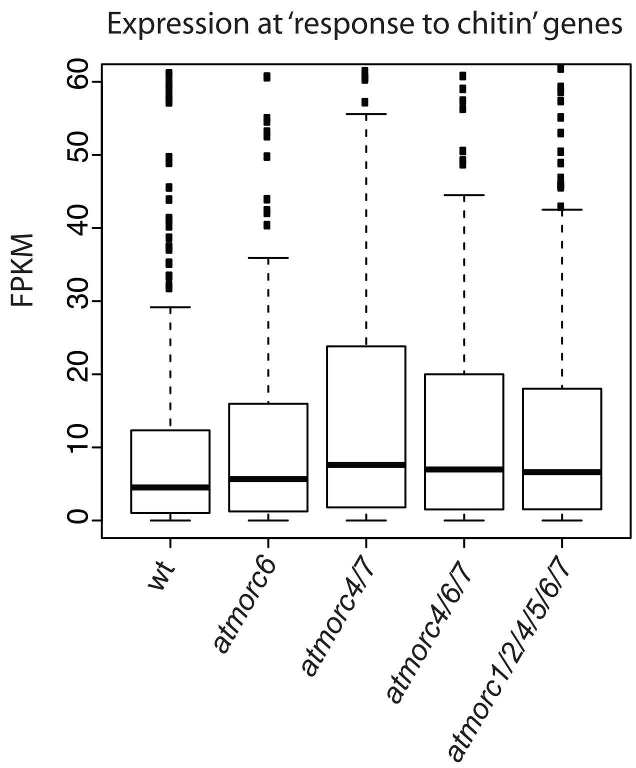

Supplement: S5 Fig — Boxplot showing FPKMs at the ‘response to chitin’ gene set (GO:0010200) in the genotypes indicated. (PDF) [file pgen.1005998.s005.pdf]
