# Supplementary material for: Arabidopsis AtMORC4 and AtMORC7 Form Nuclear Bodies and Repress a Large Number of Protein-Coding Genes
Source: PLoS Genet. 2016 May 12;12(5):e1005998. doi: 10.1371/journal.pgen.1005998 (PMC4865129; doi:10.1371/journal.pgen.1005998)

Fig. S6: Negligible DNA methylation changes genome wide and at AtMORC targets in *AtMORC* knockouts.

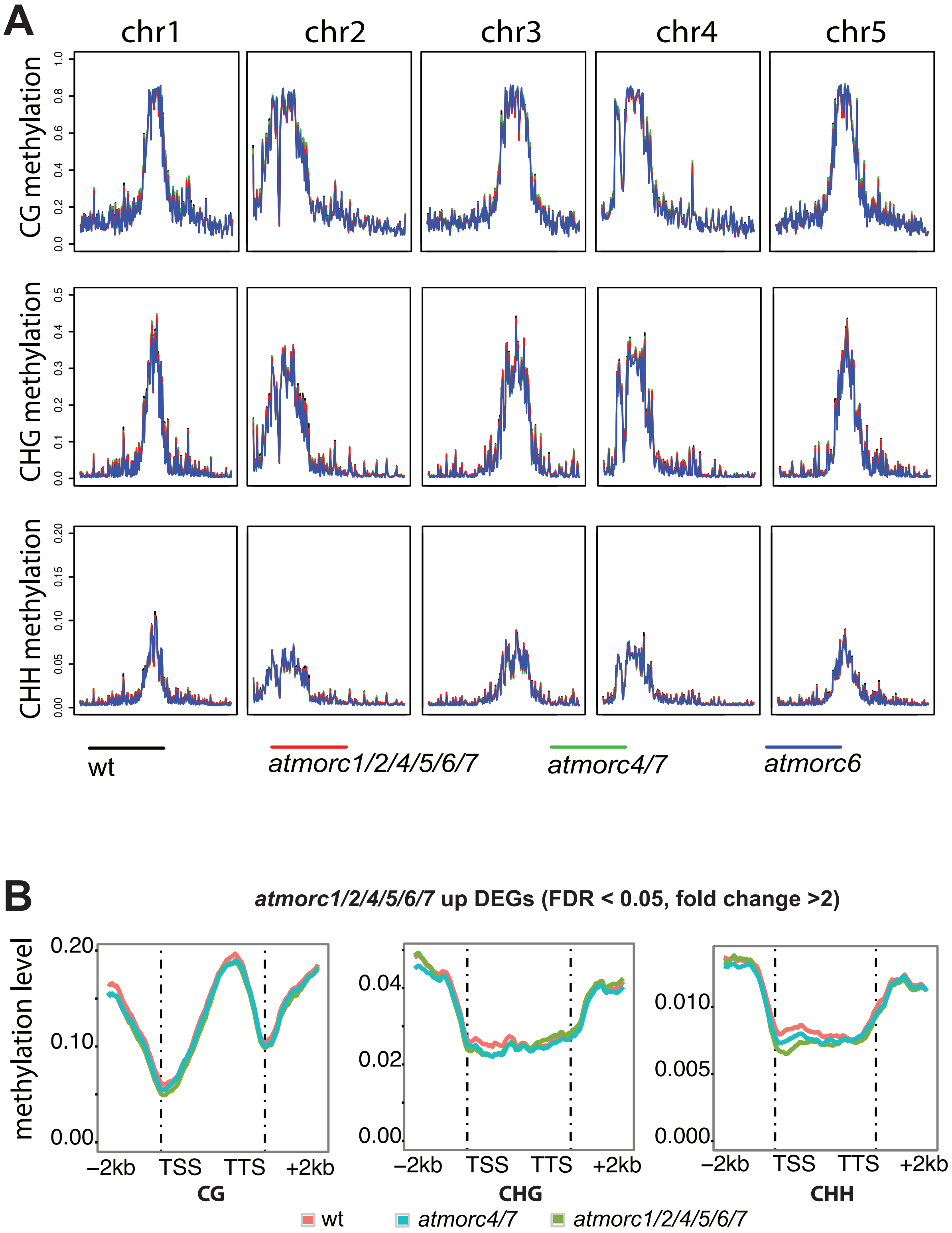

Supplement: S6 Fig — (A) Genome wide profiles of CG, CHG, and CHH context methylation in the wt, atmorc4/7, atmorc6, and atmorc1/2/4/5/6/7 backgrounds. Average of two biological replicates of each genotype, except atmorc6 (data obtained from GSE54677) [35]. (B) Metaplot of methylation levels in wt, atmorc4/7 and atmorc1/2/4/5/6/7 over DEGs (>2 fold change, FDR<0.05) in atmorc1/2/4/5/6/7 background, in CG, CHG and CHH contexts. TSS = transcriptional start site, TTS = transcriptional termination site. (PDF) [file pgen.1005998.s006.pdf]
