# Supplementary material for: Arabidopsis AtMORC4 and AtMORC7 Form Nuclear Bodies and Repress a Large Number of Protein-Coding Genes
Source: PLoS Genet. 2016 May 12;12(5):e1005998. doi: 10.1371/journal.pgen.1005998 (PMC4865129; doi:10.1371/journal.pgen.1005998)

Fig. S7: Loss of AtMORC does not significantly impact any of the major DNA methylation pathways and does not act downstream of DNA methylation.

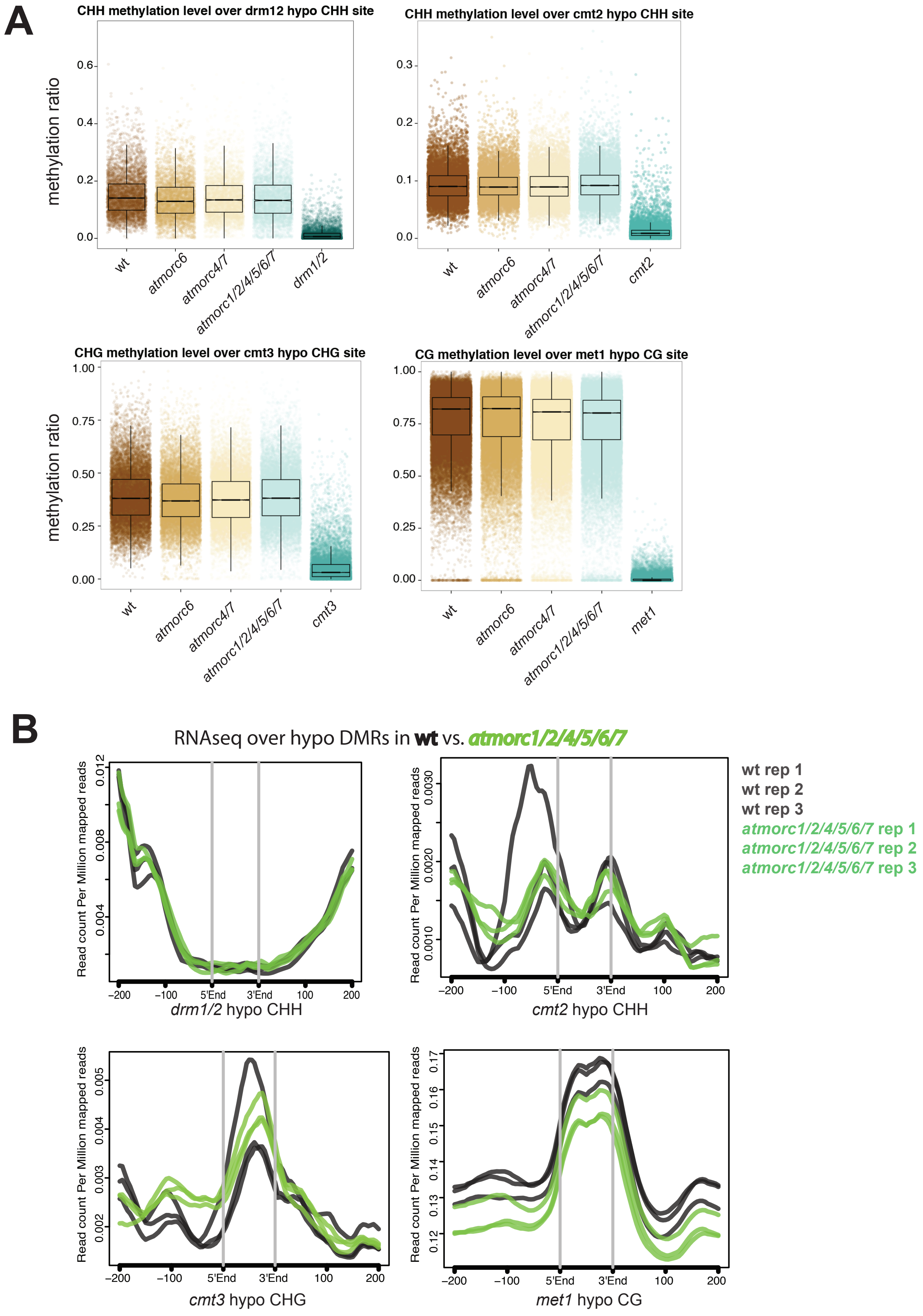

Supplement: S7 Fig — (A) Boxplots for methylation levels at drm1/2 CHH, cmt2 CHH, cmt3 CHG, and met1 CG defined hypomethylated DMRs [8,49] in the wt, atmorc4/7, atmorc6, atmorc1/2/4/5/6/7, and control methyltransferase mutant backgrounds indicated. (B) RNA-seq from wt and atmorc1/2/4/5/6/7 (black and green, respectively, three replicates each, see Fig 3) over methylated loci defined by drm1/2 CHH, cmt2 CHH, cmt3 CHG, and met1 CG hypo DMRs (as in (A)). (PDF) [file pgen.1005998.s007.pdf]
