# Supplementary material for: Arabidopsis AtMORC4 and AtMORC7 Form Nuclear Bodies and Repress a Large Number of Protein-Coding Genes
Source: PLoS Genet. 2016 May 12;12(5):e1005998. doi: 10.1371/journal.pgen.1005998 (PMC4865129; doi:10.1371/journal.pgen.1005998)
Supplement: S8 Fig — (A) Overlap of atmorc1/2/4/5/6/7 defined hypo CHH DMRs with previously defined drm1/2 and cmt2 hypo CHH DMRs [8,49]. (B) Overlap of atmorc1/2/4/5/6/7 hypo CHH DMRs with CHH loci prone to spontaneous epiallelic variation [50]. (PDF) [file pgen.1005998.s008.pdf]

Fig. S8: *atmorc1/2/4/5/6/7* hypo CHH DMRs overlap with RdDM sites.

A

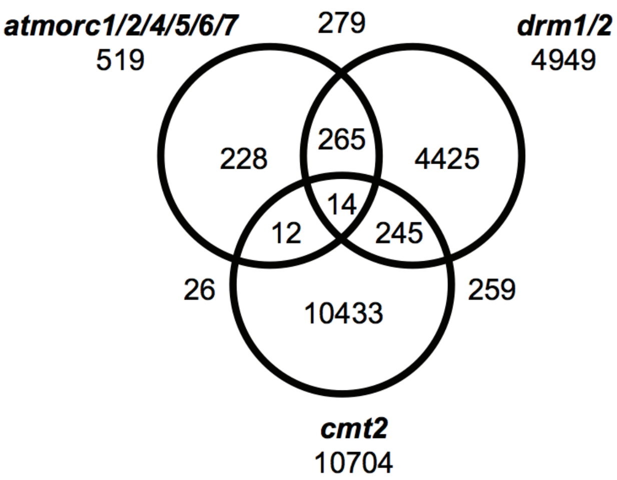

54% of *atmorc1/2/4/5/6/7* hypo CHH DMRs overlap with *drm1/2*
2% of *atmorc1/2/4/5/6/7* hypo CHH DMRs overlap exclusively with *cmt2*

B

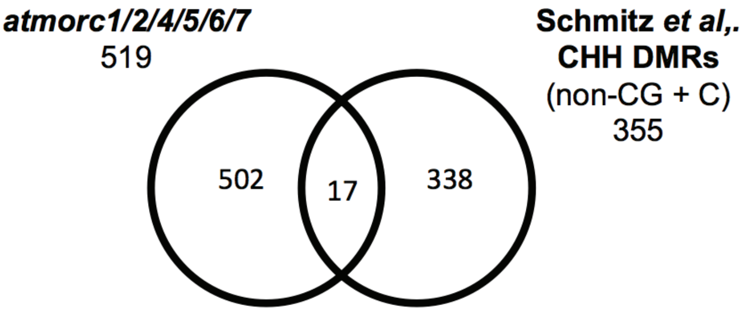

3% of *atmorc1/2/4/5/6/7* hypo CHH DMRs
overlap with spontaneous epi-alleles
