# Supplementary material for: Arabidopsis AtMORC4 and AtMORC7 Form Nuclear Bodies and Repress a Large Number of Protein-Coding Genes
Source: PLoS Genet. 2016 May 12;12(5):e1005998. doi: 10.1371/journal.pgen.1005998 (PMC4865129; doi:10.1371/journal.pgen.1005998)

Fig. S10: *atmorc1/2/4/5/6/7* hypo CHH DMRs show evidence for transcriptional de-repression.

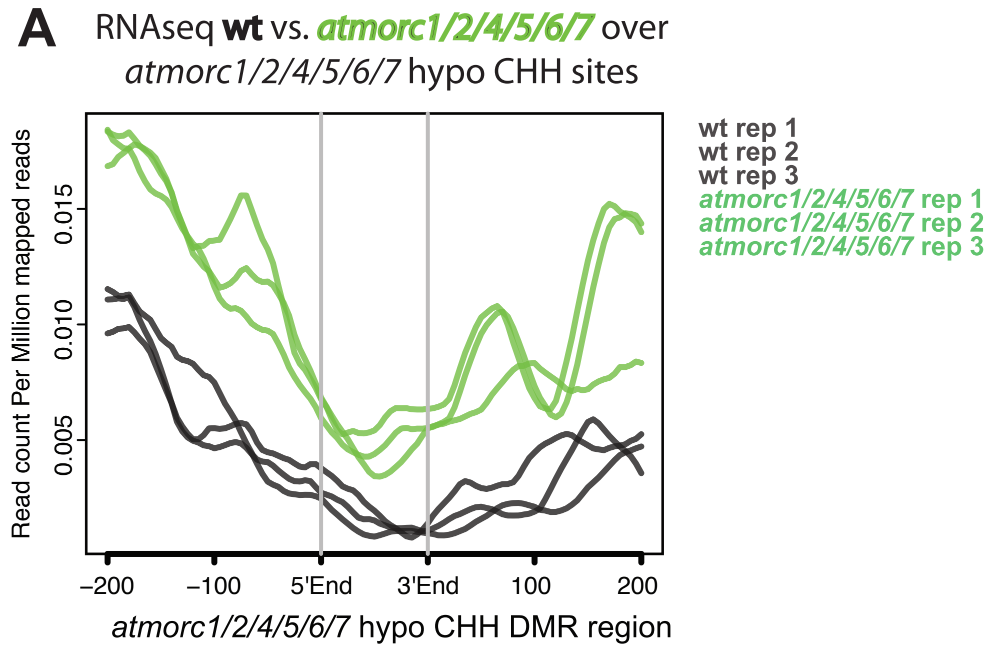

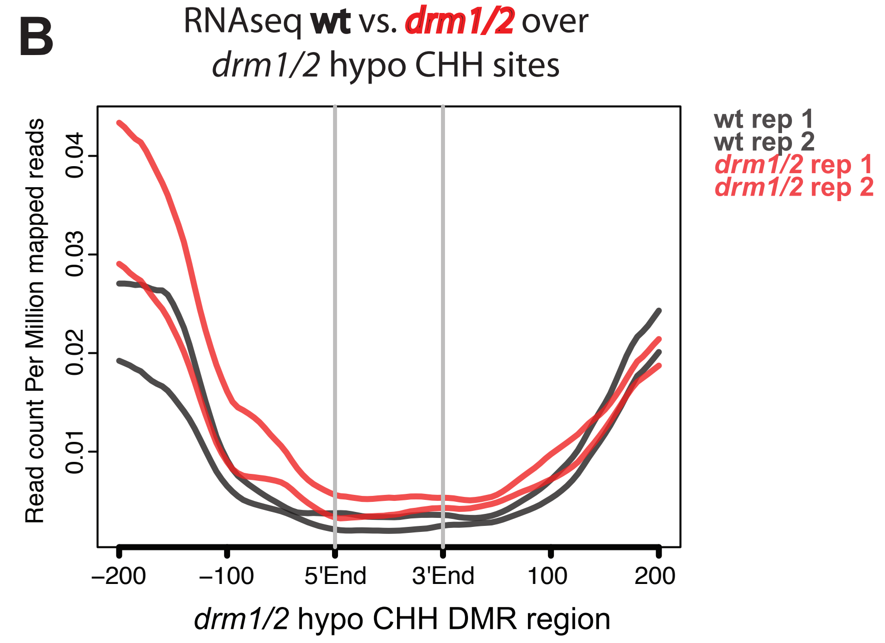

Supplement: S10 Fig — (A) RNA-seq metaplot of wt vs. atmorc1/2/4/5/6/7 (black and green, respectively, three replicates each, see Fig 3) over atmorc1/2/4/5/6/7 defined hypo CHH DMRs. (B) RNA-seq metaplot of wt vs. drm1/2 (black and red, respectively, two replicates each) over drm1/2 hypo CHH DMRs (data from GEO:GSE51304) [8]. (PDF) [file pgen.1005998.s010.pdf]
