# Supplementary material for: Arabidopsis AtMORC4 and AtMORC7 Form Nuclear Bodies and Repress a Large Number of Protein-Coding Genes
Source: PLoS Genet. 2016 May 12;12(5):e1005998. doi: 10.1371/journal.pgen.1005998 (PMC4865129; doi:10.1371/journal.pgen.1005998)
Supplement: S1 Table — List of relevant primers used in the study. (PDF) [file pgen.1005998.s013.pdf]

Table S1: Primers used in this study.

**RT-PCR primers**		
AtMu1	TAATTTGGCTGACGGAATCAC	ATTTGGGGGAAAACAAATGAG
HYPO	AACTCGGGAAAATCAGTTGCT	TTCTTCATGCCGTAAGCTGTT
SDC	AATGTAAGTTGTAAACCATTTGAACGTGACC	CAGGCATCCGTAGAACTCATGAGC
SoloLTR	AACTAACGTCATTACATACACATCTTG	AATTAGGATCTTGTTTGCCAGCTA
UNK	AAGTGGTGAGAAAGCAGAAACGAG	ACCCACTCAGCCTAACTCTACG
ROMT5	GTATCCTTTGGCCCGGTATT	GCCTCTTCGAAATGCCATAA
Actin 7	TCGTGGTGGTGAGTTTGTTAC	CAGCATCATCACAAGCATCC
morc4-1 T-DNA GK-249F08	ATGGAGCCTATCGTGAAGC	GCCACCTGCAGAAACTTCC
morc7-1 T-DNA SALK_051729	ATCCTATTCCTGCGAATCCG	CTCCATATGATTCAGACTGTGG
UBQ10	GATCTTTGCCGGAAAACAATTGGAGG	CGACTTGTCATTAGAAAGAAAGAGAT
**Genotyping primers**		
atmorc1-2 SAIL_893_B06	TTGCAGTTTGGAACCAAAATC	TGAGTTTTGACGACGATGATG
atmorc2-1 SALK_072774C	CTACTCAGAGCGTTGGCATTC	GTTGTAGCTGTATGGGGCTTG
atmorc3-2 SALK_043244	TTGTGTCCTAATGTGCTGTGG	AATCAAGCCATATGCAAATCC
atmorc4-1 GK-249F08	TCAGGAAAGATTTCACGAATTG	ACCTGCAGAAACTTCCCAATC
atmorc5-1 SALK_049050C	GTTGGGATAGATAAGGCGACC	TGTCGAGAAATCGTTCCTTTG
atmorc6-3 GABI_599B06	ACATCTTCCAATGGCTGAATC	GCTGGTGTCACTTCTTCATCC
atmorc7-1 SALK_051729	GTCGAAAGGATGTGAGAAACG	TTCCATTCAATTGCTTGGTTC
